# Optimizing anaerobic growth rate and fermentation kinetics in *Saccharomyces cerevisiae* strains expressing Calvin-cycle enzymes for improved ethanol yield

**DOI:** 10.1186/s13068-017-1001-z

**Published:** 2018-01-25

**Authors:** Ioannis Papapetridis, Maaike Goudriaan, María Vázquez Vitali, Nikita A. de Keijzer, Marcel van den Broek, Antonius J. A. van Maris, Jack T. Pronk

**Affiliations:** 1 Department of Biotechnology, Delft University of Technology, Van der Maasweg 9, 2629 HZ Delft, The Netherlands; 2 Present Address: School of Biotechnology, Division of Industrial Biotechnology, KTH Royal Institute of Technology, AlbaNova University Centre, 10691 Stockholm, Sweden

**Keywords:** Biofuels, NADH, NADPH, Redox cofactor balance, Yeast, CO_2_, Fermentation, Anaerobic metabolism

## Abstract

**Background:**

Reduction or elimination of by-product formation is of immediate economic relevance in fermentation processes for industrial bioethanol production with the yeast *Saccharomyces cerevisiae*. Anaerobic cultures of wild-type *S. cerevisiae* require formation of glycerol to maintain the intracellular NADH/NAD^+^ balance. Previously, functional expression of the Calvin-cycle enzymes ribulose-1,5-bisphosphate carboxylase (RuBisCO) and phosphoribulokinase (PRK) in *S. cerevisiae* was shown to enable reoxidation of NADH with CO_2_ as electron acceptor. In slow-growing cultures, this engineering strategy strongly decreased the glycerol yield, while increasing the ethanol yield on sugar. The present study explores engineering strategies to improve rates of growth and alcoholic fermentation in yeast strains that functionally express RuBisCO and PRK, while maximizing the positive impact on the ethanol yield.

**Results:**

Multi-copy integration of a bacterial-RuBisCO expression cassette was combined with expression of the *Escherichia coli* GroEL/GroES chaperones and expression of PRK from the anaerobically inducible *DAN1* promoter. In anaerobic, glucose-grown bioreactor batch cultures, the resulting *S. cerevisiae* strain showed a 31% lower glycerol yield and a 31% lower specific growth rate than a non-engineered reference strain. Growth of the engineered strain in anaerobic, glucose-limited chemostat cultures revealed a negative correlation between its specific growth rate and the contribution of the Calvin-cycle enzymes to redox homeostasis. Additional deletion of *GPD2*, which encodes an isoenzyme of NAD^+^-dependent glycerol-3-phosphate dehydrogenase, combined with overexpression of the structural genes for enzymes of the non-oxidative pentose-phosphate pathway, yielded a CO_2_-reducing strain that grew at the same rate as a non-engineered reference strain in anaerobic bioreactor batch cultures, while exhibiting a 86% lower glycerol yield and a 15% higher ethanol yield.

**Conclusions:**

The metabolic engineering strategy presented here enables an almost complete elimination of glycerol production in anaerobic, glucose-grown batch cultures of *S. cerevisiae*, with an associated increase in ethanol yield, while retaining near wild-type growth rates and a capacity for glycerol formation under osmotic stress. Using current genome-editing techniques, the required genetic modifications can be introduced in one or a few transformations. Evaluation of this concept in industrial strains and conditions is therefore a realistic next step towards its implementation for improving the efficiency of first- and second-generation bioethanol production.

**Electronic supplementary material:**

The online version of this article (10.1186/s13068-017-1001-z) contains supplementary material, which is available to authorized users.

## Background

Transport fuels derived from microbial fermentation combine compatibility with current combustion-engine technology with the potential to achieve lower carbon footprints than those of petrochemistry-derived fuels [1]. Bioethanol, the biofuel with the highest current global production volume (ca. 100 billion litres in 2015 [2]), is almost exclusively made via the fermentation of sugars by the yeast *Saccharomyces cerevisiae* [3, 4]. First-generation bioethanol processes, which mainly use hydrolysed corn starch or sucrose from sugar cane as feedstocks, reach high ethanol productivities and yields [5]. For example, sugar-cane-based bioethanol production in Brazil often approaches 92% of the theoretical maximum of 0.51 g g_hexose_^−1^ [6]. Since the feedstock is the largest cost contributor in first-generation industrial ethanol production [7], even modest improvements in ethanol yield can significantly improve process economics.

Carbon losses during anaerobic bioethanol production result from the formation of biomass, CO_2_, and by-products, with glycerol formation requiring up to 4% of the sugar substrate in industrial processes [2, 8]. Glycerol plays multiple roles in the physiology of *S. cerevisiae*. While sugar dissimilation via the enzymes of glycolysis and alcoholic fermentation is redox-neutral, yeast cells still need to reoxidize an ‘excess’ of NADH formed in biosynthetic reactions [9, 10]. In anaerobic cultures, which cannot reoxidize NADH by respiration, this essential role is fulfilled by NADH-dependent reduction of dihydroxyacetone-phosphate to glycerol-3-phosphate (catalysed by the isoenzymes Gpd1 and Gpd2), followed by its dephosphorylation to glycerol (catalysed by the isoenzymes Gpp1 and Gpp2) [9, 10]. Glycerol-3P, an intermediate in this pathway, also provides the glycerol backbone of glycerolipids [11, 12]. This role of glycerol-3P is, however, non-essential, since glycerolipids can also be formed from dihydroxyacetone-phosphate via the reactions catalysed by dihydroxyacetone-phosphate acyltransferase and 1-acylglycerol-3-phosphate acyltransferase [12]. Furthermore, glycerol has been identified as the major compatible solute in osmotically stressed, glucose-grown *S. cerevisiae* cultures [13, 14]. In contrast, trehalose has recently been reported to be the predominant compatible solute in ethanol-grown cultures [15].

In *S. cerevisiae*, *GPD1* is up-regulated under osmotic stress, while *GPD2* is up-regulated during anaerobiosis [16–19]. Despite their differential regulation, complete elimination of glycerol production requires deletion of both genes. Anaerobic growth of Gpd^−^ strains requires addition of external electron acceptors such as acetoin, which can be reduced to 2,3-butanediol [19]. In addition, acetate-dependent anaerobic growth of *gpd1Δ gpd2Δ S. cerevisiae* strains has been demonstrated in strains expressing an engineered pathway for NADH-linked reduction of acetate to ethanol [20]. When the decreased osmotolerance of these strains is addressed by evolutionary or targeted metabolic engineering [21, 22], this acetate reduction strategy is particularly attractive for ethanol production from lignocellulosic hydrolysates, in which acetic acid is a ubiquitous inhibitor of yeast performance [23, 24].

Several strategies have been explored to decrease glycerol production by *S. cerevisiae* in first-generation bioethanol processes, including redox engineering of ammonium assimilation [8], expression of a non-phosphorylating, NADP^+^-dependent glyceraldehyde-3-phosphate dehydrogenase [25] and reduction of biomass yields by forcing increased ATP turnover, e.g. by addition of weak organic acids to bioreactors [26, 27]. While resulting in significantly reduced glycerol yields in laboratory cultures, these strategies also led to reduced growth rates and/or depended on specific growth conditions.

In a previous study, our group functionally expressed the Calvin-cycle enzymes phosphoribulokinase (PRK) and ribulose-1,5-bisphosphate carboxylase/oxygenase (RuBisCO) in *S. cerevisiae*, thereby enabling the use of CO_2_ as alternative electron acceptor for reoxidation of cytosolic NADH [28]. Together, these enzymes convert 1 mol of the pentose-phosphate-pathway intermediate ribulose-5-phosphate and 1 mol of CO_2_ into 2 mol of 3-phosphoglycerate, thus bypassing NADH formation in glycolysis. Since CO_2_ is abundantly present in fermenting yeast cultures, implementation of this strategy is not limited by the composition of industrial media. Co-expression of a plant PRK gene (*Spinacia oleracea prk*), a bacterial RuBisCO gene (*Thiobacillus denitrificans cbbm*), and the *Escherichia coli* chaperone genes *groEL* and *groES* yielded a *S. cerevisiae* strain that displayed a 90% decrease in glycerol yield in anaerobic glucose/galactose-grown chemostat cultures and a 60% decrease in glycerol yield in anaerobic galactose-grown batch cultures [28]. These results were obtained without deletion of *GPD1* or *GPD2*, indicating that, especially in the chemostat cultures, NADH oxidation enabled by expression of the RuBisCO/PRK pathway could compete efficiently with the native glycerol pathway for NADH oxidation. Retaining a low background capacity for glycerol production is attractive for industrial applications in view of its positive impact on osmotolerance [22]. The proof-of-principle strain described in our earlier paper required galactose as a carbon source to induce gene expression, which led to low specific growth rates in batch cultures [28]. Moreover, its different performances in batch and chemostat cultures indicated that further analysis and optimization of this redox-engineering strategy is required before implementation in industry can be considered.

The goal of the present study was to investigate and address requirements for efficient carbon dioxide reduction via heterologously expressed Calvin-cycle enzymes in fast-growing anaerobic batch cultures on glucose. To this end, we used CRISPR-Cas9-mediated genome editing for integration of constitutively expressed gene cassettes for RuBisCO and PRK in the yeast genome. The performance of the constructed strains was quantitatively analysed in anaerobic glucose-limited chemostats and batch cultures. Based on the results of these analyses, additional metabolic engineering steps were implemented, yielding *S. cerevisiae* strains that displayed the full benefit of glycerol yield reduction and ethanol yield improvement in anaerobic, glucose-grown batch cultures growing at near-wild-type specific growth rates.

## Methods

### Maintenance of strains

All yeast strains used in this study (Table 1) originate from the CEN.PK lineage of *S. cerevisiae* strains [29, 30]. Cultures were propagated in synthetic medium [31] supplemented with 20 g L^−1^ glucose. Uracil (0.14 g L^−1^) was added when auxotrophic strains were propagated. *E. coli XL*-*1* blue stock cultures were grown in LB medium (5 g L^−1^ Bacto yeast extract, 10 g L^−1^ Bacto tryptone, 5 g L^−1^ NaCl), supplemented with 100 μg mL^−1^ ampicillin or 50 μg mL^−1^ kanamycin. Frozen stocks were prepared by addition of glycerol (30% v/v final concentration) to growing cultures and subsequent storage at − 80 °C.

**Table 1:** *S. cerevisiae* strains used in this study

Strain name	Relevant genotype	Parental strain	Origin
CEN.PK113-5D	*MAT*a *ura3*-*52*	–	[29]
CEN.PK122	*MAT*a/*MATα*	–	[29]
IMX585	*MAT*a *URA3 can1::cas9*-*natNT2*	CEN.PK113-7D	[34]
IMX581	*MAT*a *ura3*-*52 can1::cas9*-*natNT2*	CEN.PK113-5D	[34]
IMX673	*MAT*a/*MATα ura3*-*52/ura3*-*52 CAN1/can1Δ::cas9*-*natNT2*	CEN.PK115	[34]
IME324	*MAT*a *ura3*-*52 can1::cas9*-*natNT2* p426-*TEF* (empty)	IMX581	[22]
IMX765^a^	*MAT*a/*MAT*a *ura3*-*52*/*ura3*-*52 can1::cas9*-*natNT2*/*can1::cas9*-*natNT2 sga1::cbbm* (9 copies)*, groES, groEL*/*sga1::cbbm* (9 copies)*, groES, groEL*	IMX581	This study
IMX773	*MAT*a/*MAT*a *ura3*-*52*/*ura3*-*52 can1::cas9*-*natNT2*/*can1::cas9*-*natNT2 sga1::cbbm* (9 copies)*, groES, groEL*/*sga1::cbbm* (9 copies)*, groES, groEL X*-*2::*p*YEN1*-*prk*/*X*-*2::*p*YEN1*-*prk* pUDR164	IMX765	This study
IMX774	*MAT*a/*MAT*a *ura3*-*52*/*ura3*-*52 can1::cas9*-*natNT2*/*can1::cas9*-*natNT2 sga1::cbbm* (9 copies)*, groES, groEL*/*sga1::cbbm* (9 copies)*, groES, groEL X*-*2::*p*DAN1*-*prk*/*X*-*2::*p*DAN1*-*prk* pUDR164	IMX765	This study
IMX949	*MAT*a/*MAT*a *ura3*-*52*/*ura3*-*52 can1::cas9*-*natNT2*/*can1::cas9*-*natNT2 sga1::cbbm* (9 copies)*, groES, groEL*/*sga1::cbbm* (9 copies)*, groES, groEL X*-*2::*p*DAN1*-*prk*/*X*-*2::*p*DAN1*-*prk gpd2Δ*/*gpd2Δ* pROS10-gRNA.*GPD2*	IMX774	This study
IMX1443	*MAT*a/*MAT*a *ura3*-*52*/*ura3*-*52 can1::cas9*-*natNT2*/*can1::cas9*-*natNT2 sga1::cbbm* (9 copies)*, groES, groEL*/*sga1::cbbm* (9 copies)*, groES, groEL X*-*2::*p*DAN1*-*prk*/*X*-*2::*p*DAN1*-*prk gpd2::*p*TDH3*-*RPE1,* p*PGK1*-*TKL1,* p*TEF1*-*TAL1,* p*PGI1*-*NQM1,* p*TPI1*-*RKI1,* p*PYK1*-*TKL2*/*gpd2::*p*TDH3*-*RPE1,* p*PGK1*-*TKL1,* p*TEF1*-*TAL1,* p*PGI1*-*NQM1,* p*TPI1*-*RKI1,* p*PYK1*-*TKL2* pUDR164	IMX774	This study
IME369	*MAT*a/*MATα ura3*-*52/ura3*-*52 CAN1/can1Δ::cas9*-*natNT2* p426-*TEF* (empty)	IMX673	This study
IMX1472	*MAT*a *ura3*-*52 can1::cas9*-*natNT2 gpd2::*p*TDH3*-*RPE1,* p*PGK1*-*TKL1,* p*TEF1*-*TAL1,* p*PGI1*-*NQM1,* p*TPI1*-*RKI1,* p*PYK1*-*TKL2* pROS11-gRNA*.GPD2*	IMX581	This study
IMX1489	*MAT*a *ura3*-*52 can1::cas9*-*natNT2 gpd2::*p*TDH3*-*RPE1,* p*PGK1*-*TKL1,* p*TEF1*-*TAL1,* p*PGI1*-*NQM1,* p*TPI1*-*RKI1,* p*PYK1*-*TKL2 sga1::*p*DAN1*-*prk, cbbm* (9 copies), *groES, groEL* pUDR103	IMX1472	This study

^a^Indicates spontaneous diploidization; see “Results” section

### Plasmid and cassette construction

All plasmids used in this study are listed in Table 2. CRISPR/Cas9-based genome editing was used to perform genetic modifications in all constructed strains [32]. Unique CRISPR/Cas9 sequences targeting *GPD2*, *SGA1* or X-2 were identified using a publicly available list [32]. A list of all primers and oligonucleotides used in this study is given in Additional file 1. Phusion High-Fidelity DNA Polymerase (Thermo-Scientific, Waltham, MA) was used for PCR amplification of plasmids and expression cassettes in all cases, according to the manufacturer’s guidelines.

**Table 2:** Plasmids used in this study

Name	Characteristics	Origin
p426-*TEF*	2 μm ori, *URA3*, p*TEF1*-t*CYC1* empty vector	[38]
pMEL10	2 μm ori, *KlURA3*, p*SNR52*-gRNA.*CAN1*-*tSUP4*	[34]
pMEL11	2 μm ori, *amdS*, p*SNR52*-gRNA.*CAN1*-*tSUP4*	[34]
pROS10	*URA3*, gRNA.*CAN1*-2 μm ori-gRNA.*ADE2*	[34]
pROS11	*amdS*, gRNA.*CAN1*-2 μm ori-gRNA.*ADE2*	[34]
pUD232	Delivery vector, p*TEF1*-*groEL*-*tACT1*	[28]
pUD233	Delivery vector, p*TPI1*-*groES*-*tPGI1*	[28]
pUDE046	2 μm ori, p*GAL1*-*prk*-t*CYC1*	[28]
pBTWW002	2 μm ori, *URA3*, p*TDH3*-*cbbm*-t*CYC1*	[28]
pUD344	p*PGI1*-*NQM1*-t*NQM1* PCR template vector	[48]
pUD345	p*TPI1*-*RKI1*-t*RKI1* PCR template vector	[48]
pUD346	p*PYK1*-*TKL2*-t-*TKL2* PCR template vector	[48]
pUD347	p*TDH3*-*RPE1*–t*RPE1* PCR template vector	[48]
pUD348	p*PGK1*-*TKL1*-t*TKL1* PCR template vector	[48]
pUD349	p*TEF1*-*TAL1*-t*TAL1* PCR template vector	[48]
pUDR103	2 μm ori, *KlURA3*, p*SNR52*-gRNA.*SGA1*-t*SUP4*	[22]
pUDR119	2 μm ori, *amdS*, p*SNR52*-gRNA.*SGA1*-t*SUP4*	This study
pUDR164	2 μm ori, *KlURA3*, p*SNR52*-gRNA.*X*-*2*-t*SUP4*	This study
pJET-*cbbm*	PCR template vector for *cbbm* amplification	This study

For markerless genomic integration of gene cassettes, plasmids expressing unique gRNAs targeting the *SGA1* locus or the intergenic region X-2 [33] were constructed. The plasmid backbones of puDR119 and pURD164 were obtained by PCR amplification using the primer combination 5792–5980 and plasmids pMEL11 and pMEL10 [34], respectively, as templates. The plasmid inserts of pUDR119 and pUDR164, containing the expression cassettes coding for the unique 20-bp gRNA sequences targeting *SGA1* and X-2, respectively, were obtained by PCR amplification using the primer combinations 5979–7023 for *SGA1*, 5979–7374 for X-2, and plasmids pMEL11 and pMEL10, respectively, as templates. The assembly of plasmids pUDR119 and pUDR164 was performed in vitro using the Gibson Assembly Cloning kit (New England Biolabs, Ipswich, MA) following the supplier’s guidelines. The assembly was enabled by homologous sequences present at the 5′ and 3′ ends of the PCR-amplified plasmid backbones and inserts. In each case, 1 μL of the Gibson-assembly mix was used for *E. coli XL*-*1* blue transformation by electroporation, performed in a Gene PulserXcell Electroporation System (Biorad, Hercules, CA). Correct assembly of plasmids was confirmed by diagnostic PCR (Dreamtaq, Thermo-Scientific) or restriction digestion. The constructed plasmids pUDR119 and pUDR164 were isolated from transformed *E. coli* cultures using a Sigma GenElute Plasmid kit (Sigma-Aldrich, St. Louis, MO) and used in transformations of *S. cerevisiae*.

For markerless deletion of *GPD2*, the plasmid backbone of pROS10 (*URA3* marker) or pROS11 (*amdS* marker) was PCR amplified using primer combination 5793–5793 (double binding). The plasmid insert, containing the expression cassette coding for the unique 20-bp gRNA sequence targeting *GPD2*, was obtained using primer combination 6966–6966 (double binding) and plasmid pROS10 as template.

A yeast codon-optimized cassette for *T. denitrificans cbbm* overexpression [28] was obtained by PCR amplification using plasmid pBTWW002 as template and primer combination 7549–7550. The resulting fragment was ligated to a pJET/1.2 blunt vector (Thermo-Scientific) following the supplier’s protocol and cloned to *E. coli*. The resulting plasmid was used as PCR template to generate *cbbm* integration cassettes, using primer combinations 11206-6285, 6280–6273, 6281–6270, 6282–6271, 6284–6272, 6283–6275, 6287–6276, 6288–6277 and 6289–7075. The overexpression cassettes of *cbbm* (p*TDH3*-*cbbm*-t*CYC1*) were genetically identical, except for different overhangs present at the 5′ and 3′ ends of the fragments to allow for in vivo homologous recombination. Codon-optimized yeast expression cassettes of *groEL* (p*TEF1*-*groEL*-t*ACT1*) and *groES* (p*TPI1*-*groES*-t*PGI1*) were obtained using plasmids pUD232 and pUD233 as templates and primer combinations 7076–7077 and 7078–7079, respectively.

The genomic sequence corresponding to the constitutive promoter of *YEN1* [35] was obtained by PCR amplification with primer combination 7933–7295 and genomic DNA of IMX585 as template. The genomic sequence of the anaerobically inducible promoter of *DAN1* [35] was obtained by PCR amplification with primer combinations 7930–7931 (integration at X-2) and 7978–7931 (integration at *SGA1*), using genomic DNA of IMX585 as template.

The terminator sequence of *PGK1* was obtained by PCR amplification using primer combinations 7084–7934 (integration at X-2) and 7084–11,205 (integration at *SGA1*), using genomic DNA of IMX585 as template.

The *S. oleracea prk*-ORF was obtained by PCR amplification using primer combinations 7297–7081 (p*YEN1*-*prk* cassette construction), 7932–7081 (p*DAN1*-*prk* cassette construction), and plasmid pUDE046 as template. The various primer combinations resulted in *prk*-ORF fragments with homologous overhangs to the different promoter sequences and the terminator sequence of *PGK1*. The complete expression cassettes (p*YEN1*-*prk*-t*PGK1* and p*DAN1*-*prk*-t*PGK1*) were assembled by in vivo homologous recombination after transformation to yeast and correct assembly was verified by diagnostic PCR and Sanger sequencing (Baseclear, Leiden, The Netherlands).

An integration cassette for *RPE1* overexpression (*pTDH3*-*RPE1*-t*RPE1*) was PCR amplified using primer combination 11593-3290 and pUD347 as a template. Similarly, integration cassettes for overexpressions of *TKL1* (p*PGK1*-*TKL1*-t*TKL1*), *TAL1* (p*TEF1*-*TAL1*-t*TAL1*), *NQM1* (p*PGI1*-*NQM1*-t*NQM1*), *RKI1* (p*TPI1*-*RKI1*-t*RKI1*) and *TKL2* (p*PYK1*-*TKL2*-t-*TKL2*) were obtained by PCR amplification using primer combinations 5909–4068, 3274–3275, 3847–3276, 4691–3277 and 3283–11595, respectively, with plasmids pUD348, pUD349, pUD344, pUD345 and pUD346, respectively, as templates. The integration cassettes included overhang sequences to allow for in vivo assembly of overexpression cassettes of the complete non-oxidative pentose-phosphate pathway and integration at the *GPD2* locus.

### Yeast genome editing and strain construction

The lithium-acetate transformation protocol [36] was used for yeast transformations. Transformation mixtures were plated on synthetic medium agar plates [31] (2% Bacto Agar, BD, Franklin Lakes, NJ), supplemented with 20 g L^−1^ glucose (final concentration) in the case of transformations with plasmids expressing the *URA3* marker. In transformations with plasmids expressing the *amdS* marker, agar plates were prepared as described previously [37]. Confirmation of the desired genotypes in each case was performed by diagnostic colony PCR using Dreamtaq polymerase (Thermo-Scientific), according to the manufacturer’s guidelines (Additional file 1). Counter-selection of plasmids expressing *URA3* was performed using 5-fluoro-orotic acid (Zymo Research, Irvine, CA), following the supplier’s guidelines. Counter-selection of plasmids expressing *amdS* was performed as described previously [12].

Co-transformation of pUDR119, 9 copies of the *cbbm* expression cassette and single copies of the expression cassettes of *groEL* and *groES* to IMX581 (after plasmid recycling from the correct mutant) yielded the RuBisCO-expressing strain IMX765. Overhangs present at the 5′ and 3′ ends of the molecules allowed for in vivo assembly of the entire construct (11 fragments) and for integration at the *SGA1* locus.

Co-transformation of the p*YEN1* and p*DAN1* sequences, respectively, the *prk*-ORF and the t*PGK1* fragments, along with plasmid pUDR164 to strain IMX765 yielded strains IMX773 and IMX774. For construction of strain IMX949, in which *GPD2* was deleted, the two fragments of the gRNA-expressing plasmid (pROS10 backbone) and the repair oligo-nucleotides 6969–6970 were co-transformed to IMX774 (after recycling of pUDR164). For construction of strain IMX1443, in which *GPD2* was deleted and the genes of the non-oxidative branch of the pentose-phosphate pathway were overexpressed, the two fragments of the gRNA-expressing plasmid (pROS11 backbone), along with the integration cassettes p*TDH3*-*RPE1*-tRPE1, p*PGK1*-*TKL1*-t*TKL1,* p*TEF1*-*TAL1*-t*TAL1,* p*PGI1*-*NQM1*-t*NQM1,* p*TPI1*-*RKI1*-t*RKI1* and p*PYK1*-*TKL2*-t*TKL2*, were co-transformed to IMX774. The entire construct (6 fragments) was assembled in vivo and integrated at the *GPD2* locus. Before stocking of strain IMX1443, the *GPD2*-targeting CRISPR plasmid was recycled by counter-selection against its *amdS* marker [12].

Co-transformation of the two fragments of the *GPD2*-targeting CRISPR plasmid (pROS11 backbone) and the non-oxidative pentose-phosphate pathway integration cassettes to strain IMX581 yielded strain IMX1472. The RuBisCO/PRK-expressing strain IMX1489 was obtained by co-transformation of pUDR103, the p*DAN1*, *prk*-ORF, t*PGK1* sequences, 9 copies of the expression cassette of *cbbm* and the expression cassettes of *groEL* and *groES* (14 fragments) to strain IMX1472 (integration at the *SGA1* locus, *GPD2*-targeting CRISPR plasmid recycled).

The reference strains IME324 and IME369 were obtained by transformation of p426-*TEF* (empty) [38] to strains IMX581 and IMX673, respectively.

### Bioreactor cultivation

Physiological characterization of *S. cerevisiae* strains was performed in anaerobic batch and chemostat cultures in 2-L bioreactors (Applikon, Delft, The Netherlands), with 1-L working volume. Salt solutions were sterilized by autoclaving at 120 °C for 20 min. Glucose solutions were autoclaved separately at 110 °C for 20 min and subsequently added to the sterile salt solutions. All cultures were grown on synthetic medium with vitamins [31], supplemented with 20 g L^−1^ glucose and with sterile solutions of the anaerobic growth factors ergosterol (10 mg L^−1^) and Tween 80 (420 mg L^−1^), as well as with 0.2 g L^−1^ sterile antifoam C (Sigma-Aldrich). Anaerobic conditions were maintained by sparging of a gas mixture of N_2_/CO_2_ (90/10%, < 10 ppm oxygen) at a rate of 0.5 L min^−1^ and culture pH was maintained at 5 by automatic addition of 2 M KOH. All cultures were grown at a stirrer speed of 800 rpm and at a temperature of 30 °C. Oxygen diffusion in the bioreactors was minimized by equipping them with Norprene tubing and Viton O-rings, and evaporation was minimized by cooling of outlet gas to 4 °C.

To generate bioreactor inocula, two pre-culture shake flasks were grown in 500-mL flasks containing 100 mL synthetic medium (20 g L^−1^ glucose). Initial pH was adjusted to 6 by addition of KOH. Cultures were grown, under atmospheric air, at 30 °C and shaken at 200 rpm. In each case, initial pre-culture flasks were inoculated from frozen *S. cerevisiae* stock cultures. After incubation for 8–12 h, cultures from these flasks were used to inoculate fresh pre-culture flasks for bioreactor inoculum propagation. In all cases, bioreactors were inoculated when pre-cultures reached mid-exponential phase (OD_660_ 4–5), to a starting OD_660_ of 0.15–0.25.

### Analytical methods

Off-gas analysis, biomass dry weight measurements, HPLC analysis of culture supernatants and correction for ethanol evaporation in bioreactor experiments were performed as described previously [20]. Optical density was determined at 660 nm, using a Libra S11 spectrophotometer (Biochrom, Cambridge, United Kingdom). In batch cultures, yields of products were calculated from samples taken at mid-exponential phase (minimum of five samples), as described previously [39]. Biomass and product yields in chemostat cultures were determined from residual glucose, biomass and metabolite concentrations in steady-state cultures, analysed after rapid quenching of culture samples [40].

For calculation of degree of reduction (electron) balances in cultures, the degrees of reduction of biomass, CO_2_, NH_4_^+^ and extracellular metabolites (glucose, ethanol, glycerol, succinate, pyruvate, lactate and acetate) were defined as described in [41].

Estimations of statistical significance of differences in yields between strains were determined with two-tailed Student’s *t* tests. All values are represented as averages ± mean deviation of independent biological replicate cultures, performed at least in duplicate.

### Enzyme-activity assays

For in vitro enzyme-activity assays of PRK [28], cells (65 mL culture volume) from exponentially growing (OD_660_ 4), anaerobic shake-flask cultures (100 mL working volume in 500 mL conical shake-flasks) on glucose-containing (20 g L^-1^) synthetic medium were harvested, and cell extracts were prepared as described previously [42]. The harvesting and sonication buffer contained 100 mM Tris–HCl, 20 mM MgCl_2_·6H_2_O and 5 mM 1,4-dithiothreitol (pH 8.2). The assay mixture [43] contained 50 mM Tris–HCl (pH 8.2), 40 mM KCl, 10 mM MgCl_2_·6H_2_O, 0.15 mM NADH, 1 mM ATP, 3 mM phosphoenolpyruvate, 1 mM 1,4-dithiothreitol, 5 U of pyruvate kinase (EC 2.7.1.40, Sigma-Aldrich), 6 U of l-lactate dehydrogenase (EC 1.1.1.27, Honeywell Fluka, Bucharest, Romania) and 20, 30, 40 or 50 μL cell extract in 1 mL total volume. Reactions were started by addition of d-ribulose-5-phosphate (2.5 mM final concentration), and PRK activity was measured at 30 °C using a Hitachi 100-60 spectrophotometer, by monitoring of NADH oxidation at 340 nm over time. Protein concentrations in cell extracts were quantified using the Lowry method [44].

### Protein extraction and proteomics analysis

For proteomics analysis, cells (5 mL culture volume) were harvested from mid-exponential-phase (OD_660_ 2), anaerobic shake-flask cultures on synthetic medium (20 g L^−1^ glucose or 20 g L^−1^ galactose), washed with ice-cold MilliQ H_2_O, and subsequently stored at − 80 °C. Frozen cells were lysed using mechanical disruption in a Precellys-24 homogeniser (Bertin Technologies, Montigny-le-Bretonneux, France) in 0.5 mL cold methanol (− 20 °C, Sigma-Aldrich). The protein concentration of the disrupted cell suspension was measured using a Qubit 2.0 fluorometer (Thermo-Scientific). A total of 250 μg protein was taken from each methanol suspension, and 10 μg bovine-serum albumin was spiked to all samples for quality control. Proteins were extracted from the disrupted cell suspension using chloroform (Sigma-Aldrich) and 20% TCA (Sigma-Aldrich). The obtained protein pellet was dissolved in 100 mM NH_4_HCO_3_ buffer (pH 7) to a final concentration of 0.5 g L^−1^. In each sample, 5 μL of 500 mM Tris-(2-carboxyethyl)phosphine hydrochloride solution (TCEP, Sigma-Aldrich) was added, and samples were incubated at 55 °C for 30 min to facilitate disulphide reduction. Alkylation was performed through the addition of 5 μL of 550 mM iodoacetamide and subsequent incubation at 25 °C in the dark for 30 min.

Proteolysis was carried out overnight at 37 °C with Trypsin Gold (Promega, WI, USA), which specifically cleaves C-terminally at lysine and arginine, at an enzyme to substrate ratio of 1:25. Gradient elution of peptides was performed on a C18 (Acquity UPLC CSH C18 Column, 130 Å, 1.7 µm, 2.1 mm × 100 mm, Ultimate 3000) (Thermo-Scientific). 20 μL of injected peptides were separated using a gradient ratio of mobile phase A (99.9% water and 0.1% formic acid; VWR) to 20% B (99.9% acetonitrile and 0.1% formic acid; VWR) for 20 min, and to 50% B for 30 min (60 min total duration).

Data acquisition was carried out using a data-dependent method using a Q Exactive Plus mass spectrometer (Thermo-Scientific). The top 15 precursors were selected for tandem-MS/MS (MS2) analysis after higher-energy collisional dissociation (HCD) fragmentation. Full MS scans covering a mass range of 400–1600 were acquired at a resolution of 70,000 (at m/z 200), with a maximum fill time of 75 ms, and an automatic gain control (AGC) target value of 3 × 10^6^. MS2 scans were acquired at a resolution of 17,500 (at m/z 200), with a maximum fill time of 75 ms, and an AGC target value of 10^5^. An isolation window of 2 m/z with a fixed first mass of 110 m/z was applied in all experiments. HCD fragmentation was induced with a normalized collision energy of 27 for all peptides. Charge-state exclusion was set to ignore unassigned 1 charge. Isotope exclusion was enabled and peptide match was preferred.

All LC–MS/MS results were searched against the *S. cerevisiae* protein database, to which the amino acid sequences of the heterologous enzymes (PRK, CbbM, GroEL, GroES) were manually added, in Proteome Discoverer 1.4 Sequest HT (Thermo-Scientific). The cleavage preference of trypsin was selected, allowing for up to two missed cleavages (C-Term K/R restrict P). Dynamic modifications were set to carbamidomethyl (C), deamidation (N/Q) and oxidation (M). Precursor mass tolerance was set to 10 ppm and fragment mass tolerance to 0.6 Da. Following peptide identification, their *q* values were calculated based on a target decoy approach with a 1% false discovery rate.

### Spot plate assay

Spot plates on synthetic medium (pH 6) were prepared as described previously [45]. Sterile solutions of glucose (180 g L^−1^) and of the anaerobic growth factors ergosterol (10 mg L^−1^) and Tween 80 (420 mg L^−1^) were additionally supplemented. All plates were inoculated with serial dilutions of exponentially growing shake-flask cultures in sterile demineralized water, prepared as described above. Plates were incubated under anaerobic conditions (10% CO_2_) at 30 °C for 48 h.

### Ploidy determination by flow cytometry

For determination of yeast ploidy, ca. 10^7^ cells were harvested from mid-exponential phase shake-flask cultures on synthetic medium (20 g L^−1^ glucose), washed twice with demineralized water and stored in 70% ethanol at 4 °C. Sample preparation and staining was performed as described previously [46]. Samples were processed using a BD Accuri C6 flow-cytometer (BD Biosciences, San Jose, CA) and analysed using the FlowJo software package (Flowjo LLC, Ashland, OR).

### Genome sequencing

DNA was isolated from yeast cells harvested from shake-flask cultures of strain IMX774 on synthetic medium (20 g L^−1^ glucose) using a Qiagen Blood & Cell Culture DNA kit (Qiagen, Germantown, MD), following manufacturer’s specifications. Paired-end sequencing (22 mln reads) was performed on a 350-bp PCR-free insert library using an Illumina HiSeq PE150 sequencer (Novogene Company Limited, Hong Kong) with a sample size of 3.3 Gb, accounting for a total coverage of 275×. Sequence data was mapped to the CEN.PK113-7D genome [30], to which the sequences of the p*DAN1*-*prk*-t*PGK1*, p*TDH3*-*cbbm*-t*CYC1*, p*TEF1*-*groEL*-t*ACT1*, and p*TPI1*-*groES*-t*PGI1* cassettes were manually added. Data processing and chromosome copy number analysis were carried out as described previously [47–51].

## Results

### Impact of PRK expression levels on in vivo CO_2_ reduction via the RuBisCO pathway in glucose-grown batch cultures

In the engineered strain used for the first demonstration of the effect of expression of the Calvin-cycle enzymes RuBisCO and PRK on the anaerobic physiology of *S. cerevisiae*, the coding sequence of *S. oleracea prk* was placed under the control of the galactose-inducible *GAL1* promoter [28]. Use of galactose as an inducer of gene expression in *S. cerevisiae* is, however, not a realistic option in large-scale industrial fermentations for ethanol production due to the price of galactose and repression of the *GAL1* promoter by glucose [52]. Furthermore, this strain expressed the *T. denitrificans* RuBisCO gene *cbbm*, as well as the *E. coli* chaperones *groEL*/*groES*, from a centromeric plasmid. Expression from plasmids with auxotrophic markers limits applicability in industrial processes [53] and the use of a centromeric vector restricted the number of *cbbm*-cassettes per cell to 1–2 [54]. The low RuBisCO activity in cell extracts of strain IMU033 [4.6 ± 0.3 nmol (mg protein)^−1^ min^−1^] [28] suggested that introduction of additional copies of the *cbbm* cassette might be relevant for improved strain performance.

In vivo tandem assembly by homologous recombination and CRISPR-mediated targeted integration at a single locus was previously shown to be an effective way to introduce multiple copies of expression cassettes without the use of multi-copy plasmids [48, 55]. To construct a galactose-independent RuBisCO-expressing platform strain with an increased number of *cbbm* cassettes, nine copies of a *cbbm* overexpression cassette, along with single expression cassettes of *groEL*/*groES,* were first integrated at the *SGA1* locus of IMX581 using CRISPR/Cas9 single-step transformation and assembly [34], yielding strain IMX765. Since high-level expression of heterologous PRK in microbes has been previously shown to be toxic [56, 57], two expression cassettes were constructed, in which the *prk* open reading frame was either placed under the control of p*YEN1* (low-level constitutive expression under a wide range of cultivation conditions, [35]) or under the control of p*DAN1* (medium-level expression induced under anaerobic conditions, [35]). These expression cassettes were integrated at the X-2 locus [33] of strain IMX765, yielding strains IMX773 and IMX774, respectively.

**Fig. 1:**
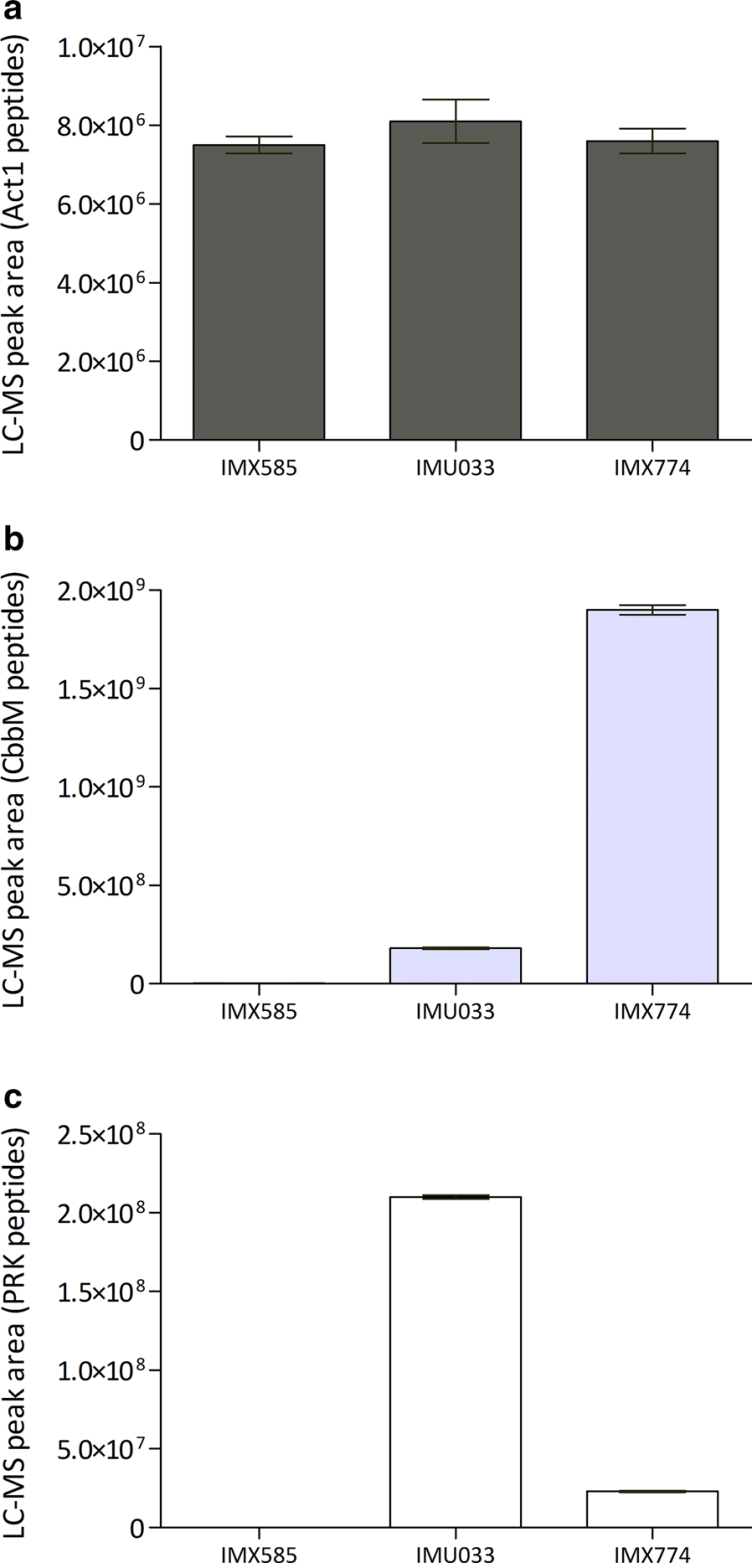
Peptide abundance in cells harvested from mid-exponential anaerobic shake-flask cultures of strains IMX585 (CEN.PK reference), IMU033 (p*GAL1*-*prk cbbm*), and IMX774 (p*DAN1*-*prk cbbm*), displayed as the sum of LC–MS peak areas of unique peptides identified per protein. **a** Act1 (internal control); **b** CbbM; **c** PRK. Cultures of IMX585 and IMX774 were grown on 20 g L^−1^ glucose (initial pH 6); cultures of IMU033 were grown on 20 g L^−1^ galactose (initial pH 6). Values represent averages ± mean deviations of measurements on independent duplicate cultures

Enzyme-activity assays in cell extracts of anaerobic, glucose-grown shake-flask cultures of strains IMX773 and IMX774 showed PRK activities of 0.14 ± 0.01 and 0.68 ± 0.33 μmol (mg protein)^−1^ min^−1^, respectively. These activities were 100- and 20-fold lower than previously measured in cell extracts of strain IMU033 under galactose-induced conditions [14.4 ± 1.5 μmol (mg protein)^−1^ min^−1^] [28]. Analysis of protein abundance of RuBisCO and PRK in strains IMU033 (p*GAL1*-*prk cbbm*) and IMX774 revealed tenfold higher CbbM levels and ninefold lower PRK levels in the latter, newly engineered strain (Fig. 1).

To investigate the effect of PRK and RuBisCO expression on the physiology of glucose-grown *S. cerevisiae*, growth and metabolite formation of strains IME324 (congenic reference strain not expressing Calvin-cycle enzymes or *E. coli* chaperones), IMX773 (p*YEN1*-*prk cbbm*) and IMX774 (p*DAN1*-*prk cbbm*) were analysed in anaerobic bioreactor batch cultures on 20 g L^−1^ glucose (Table 3; Additional file 2). In these cultures, the maximum specific growth rates of strains IMX773 and IMX774 were 13 and 31% lower, respectively, than that of the reference strain IME324 (Table 3). The lower specific growth rate of the engineered *S. cerevisiae* strains overexpressing PRK, RuBisCO and GroEL/GroES might reflect a metabolic burden resulting from increased protein synthesis (Fig. 1) [58]. This interpretation is consistent with the observation that biomass yields on glucose of strains IMX774 and IME324 were the same, even though stoichiometric analyses predicted that use of the RuBisCO/PRK pathway can lead to an up to 13.5% higher biomass yield [28]. Comparison of PRK activities in cell extracts and specific growth rates (Table 3) of strains IMX773 and IMX774 suggested that, in particular, high-level expression of PRK might have negatively affected the specific growth rate. Toxicity of high-level PRK expression is consistent with observations on galactose-grown cultures of *S. cerevisiae* IMU033 (p*GAL1*-*prk cbbm*) [28] and on PRK overexpression in *E. coli* [56, 57].

**Table 3:** Specific growth rate (μ), yields (Y) of biomass, ethanol and glycerol on glucose and stoichiometric relationships between glycerol production and biomass formation in anaerobic bioreactor batch cultures of *S. cerevisiae* strains carrying different genetic modifications

Strain	IME324	IMX773	IMX774	IMX949	IMX1443
Relevant genotype	*GPD1 GPD2*	*GPD1 GPD2* p*YEN1*-*prk* *cbbm*	*GPD1 GPD2* p*DAN1*-*prk* *cbbm*	*GPD1 gpd2Δ* p*DAN1*-*prk* *cbbm*	*GPD1 gpd2Δ* p*DAN1*-*prk* *cbbm* non-ox PPP↑
μ (h^−1^)	0.32 ± 0.02	0.28 ± 0.01*	0.22 ± 0.02**	0.22 ± 0.01**	0.30 ± 0.01
Y biomass/glucose (g g^−1^)	0.090 ± 0.002	0.089 ± 0.001	0.087 ± 0.004	0.095 ± 0.004	0.099 ± 0.005*
Y ethanol/glucose (g g^−1^)	0.364 ± 0.015	0.385 ± 0.002	0.400 ± 0.006**	0.411 ± 0.002**	0.419 ± 0.001**
Y glycerol/glucose (g g^−1^)	0.101 ± 0.003	0.098 ± 0.000	0.070 ± 0.005**	0.038 ± 0.001**	0.013 ± 0.000**
Glycerol produced/biomass [mmol (g biomass)^−1^]	12.239 ± 0.095	11.880 ± 0.008*	7.622 ± 0.409**	4.314 ± 0.245**	1.507 ± 0.119**

Cultures were grown on synthetic medium containing 20 g L^−1^ glucose (pH 5). Specific growth rates and stoichiometries were calculated from multiple sample points in the mid-exponential growth phase. Values represent averages ± mean deviations of measurements on independent cultures. Cultures of IME324, IMX949, and IMX1443 were performed in triplicate. Cultures of IMX774 were performed in quadruplicate and cultures of IMX773 were performed in duplicate. * (*p* < 0.05) and ** (*p* < 0.01) denote statistical significance of value differences between IME324 and each engineered strain in Student’s *t* tests. Degree of reduction balances constructed over the exponential growth phase yielded electron recoveries between 96 and 101%

Strain IMX773, in which *prk* was expressed from the weak constitutive *YEN1* promoter, did not show significant differences in glycerol or ethanol yields relative to the reference strain IME324 (Table 3). This result confirms that functional expression of PRK is essential for the use of CO_2_ as an electron acceptor for NADH oxidation by the engineered *S. cerevisiae* strains. In contrast, strain IMX774, which expressed *prk* from the anaerobically induced, medium-strength p*DAN1* promoter, exhibited a 31% lower glycerol yield and a 10% higher ethanol yield than the reference strain (Table 3). Furthermore, the glycerol production per gram biomass of strain IMX774 was 38% lower than that of the reference strain (Table 3). These observations indicated that the engineered PRK/RuBisCO pathway significantly contributed to NADH oxidation in anaerobic cultures of this engineered strain.

### The impact of the RuBisCO pathway on NADH oxidation is negatively correlated with the specific growth rate

The reduced glycerol yield of strain IMX774 (p*DAN1*-*prk cbbm*) in anaerobic, glucose-grown bioreactor batch cultures resembled the change in glycerol yield that was previously observed in similar galactose-grown cultures of strain IMU033 (p*GAL1*-*prk cbbm*) [28]. However, the observed reduction in glycerol yield relative to the reference strain IME324, which did not express RuBisCO or PRK, was still far from the 90% reduction that was previously observed in sugar-limited anaerobic chemostat cultures of strain IMU033, grown at a dilution rate of 0.05 h^−1^ on glucose/galactose mixtures [28]. Specific growth rates in the batch cultures were much higher than in those in chemostat cultures (Table 3, [28]). To investigate a possible relation between specific growth rate and relative contribution of the engineered PRK/RuBisCO pathway to NADH oxidation, growth and product formation of strains IMX774 (p*DAN1*-*prk cbbm*) and IME324 (reference) were analysed in anaerobic, glucose-limited chemostat cultures grown at dilution rates of 0.05 and 0.15 h^−1^ (Table 4; Additional file 2).

**Table 4:** Yields (Y) of biomass and ethanol on glucose in anaerobic chemostat cultures of *S. cerevisiae* reference strain IME324 and the RuBisCO/PRK-expressing strain IMX774

Strain	IME324		IMX774	
Relevant genotype	*GPD1 GPD2*		*GPD1 GPD2* p*DAN1*-*prk cbbm*	
Dilution rate (h^−1^)	0.05	0.15	0.05	0.15
Y biomass/glucose (g g^−1^)	0.083 ± 0.001	0.087 ± 0.007	0.082 ± 0.002	0.086 ± 0.002
Y ethanol/glucose (g g^−1^)	0.421 ± 0.001	0.411 ± 0.006	0.451 ± 0.001*^,#^	0.432 ± 0.001*^,#^

Cultures were grown on synthetic medium containing 20 g L^−1^ glucose (pH 5). Values represent averages ± mean deviations of measurements on independent duplicate cultures. * (*p* < 0.05) denotes statistical significance of differences between strains IME324 and IMX774 at the same dilution rate and ^#^ (*p* < 0.01) indicates statistical significance of differences between analyses at different dilution rates in cultures of the same strain in Student’s *t* tests. Degree of reduction balances of steady-state analyses yielded electron recoveries between 99 and 101%

**Fig. 2:**
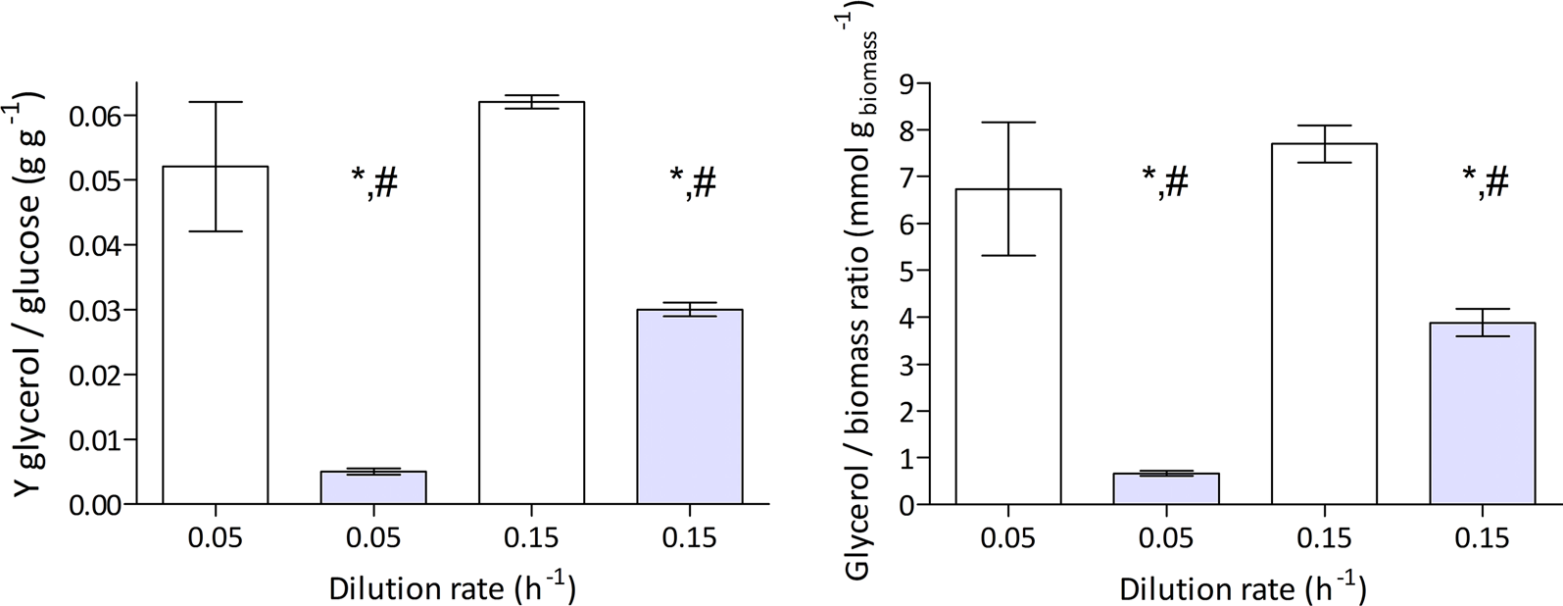
Yields (Y) of glycerol on glucose and stoichiometric relationships between glycerol production and biomass formation in anaerobic chemostat cultures of *S. cerevisiae* reference strain IME324 (white bars) and the RuBisCO/PRK-expressing strain IMX774 (p*DAN1*-*prk cbbm*, blue bars). Cultures were grown on synthetic medium containing 20 g L^−1^ glucose (pH 5). Values represent averages ± mean deviations of measurements on independent duplicate cultures. * (*p* < 0.05) denotes statistical significance of value differences between IME324 and IMX774 at the same dilution rate and ^#^ (*p* < 0.01) indicates statistical significance of differences between the two dilution rates in cultures of the same strain in Student’s *t* tests

At a dilution rate of 0.05 h^−1^, strain IMX774 showed glycerol and ethanol yields on glucose of 0.005 and 0.451 g g^−1^, respectively. These yields were 90% lower and 7% higher, respectively, than in chemostat cultures of the reference strain IME324 grown at the same dilution rate (Table 4; Fig. 2). In these slow-growing chemostat cultures, the glycerol production per gram biomass of strain IMX774 was only 0.66 mmol (g biomass)^−1^, which was 90% lower than observed in cultures of strain IME324 (Fig. 2). These results indicate that, at this low specific growth rate, the RuBisCO pathway almost completely replaced reoxidation of ‘excess’ NADH via glycerol formation, in agreement with previous results on IMU033 glucose/galactose-grown chemostat cultures on the same dilution rate [28].

The reference strain IME324 showed no significant differences in glycerol yield on glucose or in glycerol production per gram biomass when grown at a dilution rate of either 0.05 or 0.15 h^−1^ in anaerobic, glucose-limited chemostat cultures (Fig. 2). In contrast, strain IMX774 showed a fivefold higher glycerol yield on glucose and glycerol production per gram biomass when grown at a dilution rate of 0.15 h^−1^ than in cultures grown at 0.05 h^−1^ (Fig. 2). The glycerol production per gram biomass of strain IMX774 at 0.15 h^−1^ [3.88 mmol (g biomass)^−1^] was only 50% lower than that of strain IME324 grown at the same dilution rate (Fig. 2). These results demonstrated that, in strain IMX774, higher specific growth rates, which coincided with a higher glycolytic flux, resulted in a smaller contribution of the engineered PRK/RuBisCO pathway to NADH reoxidation, thereby reducing its beneficial impact on (by)product formation.

### Deletion of *GPD2* improves CO_2_ reduction to ethanol in anaerobic batch cultures of RuBisCO/PRK-expressing *S. cerevisiae*

The lower impact of RuBisCO/PRK expression​ on product formation at high specific growth rates identified the glycerol-3-phosphate dehydrogenases Gpd1 and Gpd2 as potential engineering targets for increasing the contribution of the engineered PRK/RuBisCO pathway to NADH reoxidation. Deletion of *GPD2* was previously reported to decrease glycerol formation in other engineered *S. cerevisiae* strains, without affecting osmotolerance [16–19, 22]. *GPD2* was therefore deleted in strain IMX774 (*GPD1 GPD2* p*DAN1*-*prk cbbm*), yielding strain IMX949 (*GPD1 gpd2Δ* p*DAN1*-*prk cbbm*). This deletion did not affect the specific growth rate in anaerobic bioreactor batch cultures grown on 20 g L^−1^ glucose (Table 3). Since deletion of *GPD2* has a strong negative effect on anaerobic growth of wild-type *S. cerevisiae* in the absence of an external electron acceptors [59, 60], this result further supported our conclusion that the RuBisCO pathway can effectively contribute to redox cofactor balancing in fast-growing anaerobic *S. cerevisiae* cultures. In these anaerobic bioreactor batch cultures, the glycerol and ethanol yields on glucose of strain IMX949 were 62% lower and 13% higher, respectively, than those of the reference strain IME324 (*GPD1 GPD2*) (Table 3). Furthermore, glycerol production per gram biomass of strain IMX949 was 65 and 43% lower than that of strains IME324 and IMX774, respectively (Table 3). These results clearly indicated that deletion of *GPD2* enables a higher contribution of the engineered PRK/RuBisCO pathway to anaerobic NADH reoxidation in engineered *S. cerevisiae* strains.

### Optimization of precursor supply to the RuBisCO pathway further decreases glycerol yield and enables wild-type specific growth rates in anaerobic cultures

In *S. cerevisiae*, the substrate of PRK, ribulose-5-phosphate, can be formed either by NADPH-generating oxidative decarboxylation of 6-phosphogluconate, or from glyceraldehyde-3-phosphate and fructose-6-phosphate via the re-arrangement reactions of the non-oxidative pentose-phosphate pathway (PPP, [28]). If ribulose-5-phosphate used in the RuBisCO pathway were exclusively derived from 6-phosphogluconate, this would cause an NADPH/NADP^+^ imbalance when the RuBisCO pathway completely replaces glycerol formation [10]. While formation of ribulose-5-phosphate via the non-oxidative PPP does not present such a redox constraint, extensive research on metabolic engineering of *S. cerevisiae* for pentose fermentation indicates that this pathway has a limited capacity in wild-type strains [61–64].

To test if the fermentation performance of strain IMX949 (*GPD1 gpd2Δ* p*DAN1*-*prk cbbm*) could be further improved by optimization of the ribulose-5-phosphate supply, overexpression cassettes for the non-oxidative PPP genes *RPE1*, *TKL1*, *TAL1*, *NQM1*, *RKI1* and *TKL2* were simultaneously integrated at the *GPD2* locus of IMX774, yielding strain IMX1443 (*GPD1 gpd2Δ* non-ox PPP↑ p*DAN1*-*prk cbbm*). In anaerobic, glucose-grown bioreactor batch cultures, grown under identical conditions to the previously discussed RuBisCO/PRK-expressing strains, the specific growth rate of strain IMX1443 was virtually identical to that of the reference strain IME324 (*GPD1 GPD2*) and 36% higher than that of its parental strain IMX774 (*GPD1 GPD2* p*DAN1*-*prk cbbm*) and of strain IMX949 (Table 3; Additional file 2). Furthermore, strain IMX1443 showed a 9% higher biomass yield on glucose than the reference strain IME324 (Table 3), which closely corresponds to the maximum theoretical increase for a RuBisCO/PRK-expressing strain of 13.5% [28]. These observations showed that the reduced growth rates of strains IMX774 and IMX949 were not primarily caused by accumulation of ribulose-1,5-bisphosphate or ATP depletion, resulting from an imbalance of the in vivo activities of PRK and RuBisCO. Instead, they indicate that the reduced growth rates of these strains resulted from a reduced intracellular pool of ribulose-5-phosphate, which is a key precursor for the formation of the PPP-derived biosynthetic building blocks ribose-5-phosphate and erythrose-4-phosphate.

The glycerol yield on glucose of strain IMX1443 (*GPD1 gpd2Δ* non-ox PPP↑ p*DAN1*-*prk cbbm*) was 81 and 87% lower than the yields of its parental strain IMX774 (*GPD1 GPD2* p*DAN1*-*prk cbbm*) and of the reference strain IME324 (*GPD1 GPD2*), respectively (Table 3). Consistent with an almost complete replacement of redox cofactor balancing via glycerol production by the RuBisCO pathway, its glycerol production per gram biomass was 88% lower than that of strain IME324 and closely matched the phenotype observed in slow-growing glucose-limited chemostat cultures of strain IMX774 (Table 4). Furthermore, the ethanol yield on glucose of strain IMX1443 was 15 and 5% higher than that of the reference strain IME324 and of its parental strain IMX774, respectively. The phenotype of strain IMX1443 thereby approaches the theoretical maximum benefits in glycerol reduction and increased ethanol yield, without a reduction of its specific growth rate in anaerobic, glucose-grown batch cultures. Further, the osmotolerance of strain IMX1443 was not impacted by these modifications, as shown by plate growth tests on high osmolarity (1 M glucose) medium (Additional file 3).

### The physiological benefit of RuBisCO/PRK-expression in *S. cerevisiae* is independent of strain ploidy

In the context of another study, the ploidy of strains IMX765, IMX773, and IMX774 was analysed by flow cytometry. Surprisingly, strain IMX765, the parental strain of all RuBisCO/PRK-expressing strains constructed in this study, was found to have undergone a whole-genome duplication (Additional file 4). To determine whether this diploidization was accompanied by any other chromosomal copy number variations or rearrangements, the genome of strain IMX774 (*GPD1 GPD2* p*DAN1*-*prk cbbm*) was sequenced and compared to that of the haploid congenic reference strain CENPK.113-7D [30]. This analysis showed that a ‘clean’ genome duplication had occurred, without chromosomal or segmental aneuploidies (Additional file 5).

The differences in glycerol and ethanol yields between strains IME324 (haploid, *GPD1 GPD2* reference) and IMX774 (diploid, *GPD1 GPD2* p*DAN1*-*prk cbbm*) were not expected to be influenced by ploidy variation, as biomass formation and requirements for NADH oxidation are stoichiometrically linked and the biomass yields on glucose of these strains were not significantly different (Table 3). However, as ploidy variation might affect the growth rate [65], two additional strains were constructed to investigate whether ploidy differences affected the interpretation of our results.

**Fig. 3:**
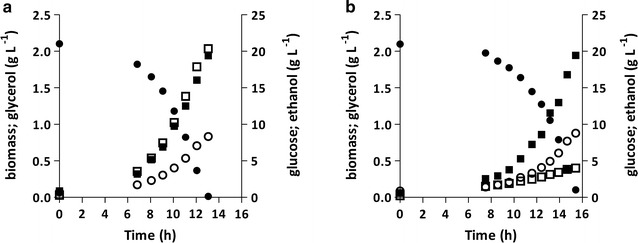
Growth, glucose consumption and product formation in anaerobic bioreactor batch cultures of *S. cerevisiae* strains IME324 (*GPD1 GPD2*) (**a**) and IMX1489 (*GPD1 gpd2Δ* p*TDH3*-*RPE1,* p*PGK1*-*TKL1,* p*TEF1*-*TAL1,* p*PGI1*-*NQM1,* p*TPI1*-*RKI1,* p*PYK1*-*TKL2* p*DAN1*-*prk cbbm*) (**b**). Cultures were grown on synthetic medium containing 20 g L^−1^ glucose (pH 5). Symbols: black circle, glucose; black square, biomass; white square, glycerol; white circle, ethanol. Representative cultures of independent duplicate experiments are shown

Strain IME369 was constructed by transformation of p426-*TEF* (empty) to IMX673, thereby generating a new diploid reference strain (*GPD1*/*GPD1 GPD2*/*GPD2*). In addition, the genetic modifications introduced in the best performing RuBisCO/PRK-expressing strain (IMX1443) were reconstructed in a haploid background, resulting in strain IMX1489 (Additional file 4). Anaerobic growth of both strains was analysed in bioreactor batch cultures, under the same conditions used for the other strains analysed in this study (Table 5; Additional file 2). The new diploid reference strain IME369 showed no significant differences in specific growth rate, biomass or ethanol yields on glucose or glycerol production per gram biomass when compared to the haploid reference strain IME324 (*GPD1 GPD2*) (Tables 3 and 5). Furthermore, the specific growth rates of the engineered strains IMX1489 (haploid) and IMX1443 (diploid) were the same, while their biomass and product yields also very closely corresponded (Tables 3 and 5, Fig. 3). Similarly to strain IMX1443, the osmotolerance of IMX1489 did not differ from that of a *GPD1 GPD2* reference strain (Additional file 3). These results indicate that the impact of the engineering strategy presented in this study does not differ between haploid and diploid *S. cerevisiae* strains.

**Table 5:** Specific growth rates (μ), yields (Y) of biomass, ethanol and glycerol on glucose and stoichiometric relationships between glycerol production and biomass formation in anaerobic bioreactor batch cultures of *S. cerevisiae* strains IME369 and IMX1489

Strain	IME369	IMX1489
Relevant genotype	*GPD1 GPD2*	*GPD1 gpd2Δ* p*DAN1*-*prk* *cbbm* non-ox PPP↑
μ (h^−1^)	0.31 ± 0.00	0.30 ± 0.01
Y biomass/glucose (g g^−1^)	0.091 ± 0.009	0.096 ± 0.001*
Y ethanol/glucose (g g^−1^)	0.376 ± 0.005	0.421 ± 0.002*
Y glycerol/glucose (g g^−1^)	0.107 ± 0.004	0.014 ± 0.000**
Glycerol produced/biomass [mmol (g biomass)^−1^]	12.189 ± 1.080	1.669 ± 0.082**

Cultures were grown on synthetic medium containing 20 g L^−1^ glucose (pH 5). Specific growth rates and stoichiometries were calculated from sample points during the mid-exponential growth phase. Values represent averages ± mean deviations of measurements on independent duplicate cultures. * (*p* < 0.02) and ** (*p* < 0.01) denote statistical significance of differences between IME324 (Table 3) and strains IME369 and IMX1489 in Student’s *t* tests. Degree of reduction balances constructed over the exponential growth phase yielded electron recoveries between 96 and 100%

## Discussion

Fixation of CO_2_ via the Calvin-cycle enzymes RuBisCO and phosphoribulokinase (PRK) plays a key role in the biological carbon cycle [66]. The Calvin cycle’s role in carbon fixation by photo- and chemoautotrophs is well established and its improvement remains a major target of research [67, 68]. In addition, in nature as well as in engineered industrial microorganisms, Calvin-cycle enzymes can increase the flexibility of intracellular redox cofactor balancing in chemoorganoheterotrophs [69, 70].

Here, we present a metabolic engineering strategy, based on expression of Calvin-cycle enzymes for redox cofactor balancing in *S. cerevisiae* [28], which enabled a near-complete elimination of glycerol production in anaerobic, glucose-grown batch cultures, with an associated increase in ethanol yield. In addition to multi-copy chromosomal integration of expression cassettes for *T. denitrificans cbbm*, this strategy encompassed expression of the *E. coli* chaperone genes *groEL* and *groES* [28], expression of the spinach *prk* gene from the anaerobically inducible *DAN1* promoter, deletion of *GPD2* and overexpression of the *S. cerevisiae* structural genes for the enzymes of the non-oxidative pentose-phosphate pathway.

A high specific growth rate of industrial *S. cerevisiae* strains is important in view of its impact on volumetric productivity and competition with microbial contaminants [2, 71]. Many previously reported redox engineering strategies for decreasing glycerol formation in *S. cerevisiae* resulted in reduced specific growth rates or requirements for specific media [8, 20, 59, 72]. Reduced growth rates of metabolically engineered microorganisms are often attributed to the metabolic burden caused by high-level expression of heterologous and/or homologous proteins [58, 73]. Despite the high-level expression of RuBisCO and yeast PPP-enzymes, the specific growth rates of haploid and diploid engineered *S. cerevisiae* strains in anaerobic batch cultures in this study were the same as those of non-engineered reference strains.

Previous research had already shown that co-expression of the *E. coli* chaperones GroEL and GroES is required for functional heterologous expression of CbbM in *S. cerevisiae* [28]. Functional expression of a plant RuBisCO in *E. coli* was also recently shown to require co-expression of no fewer than five plant chaperones [74], highlighting the importance of expression of folding-assisting proteins in the formation of functional RubisCO complexes. In the case of GroEL and GroeS specifically, it was recently shown that their expression facilitates functional expression of several heterologous proteins in yeasts [28, 75, 76]. Further, their expression can potentially be beneficial in improving strain robustness against industrial fermentation conditions [77]. In the present study, their expression may have contributed to the apparent absence of a metabolic burden in the engineered strains, by preventing cellular stress and increased protein-turnover caused by incorrect protein folding. Since multi-copy integration of expression cassettes for the form-II RuBisCO CbbM supported wild-type growth rates in anaerobic glucose-grown cultures, its replacement by an alternative RuBisCO with superior catalytic properties [68, 78] is not necessary in this experimental context. The high *K*_*m*_ of RuBisCO for CO_2_ [67] implies that microorganisms that heterologously express Calvin-cycle enzymes require high CO_2_ concentrations in the cultures for in vivo pathway activity [28, 70]. Since industrial ethanol production processes very rapidly become CO_2_ saturated, implementation of this redox engineering strategy in industry does not impose specific requirements on process design or medium composition [8].

As recently demonstrated in engineered *E. coli* strains expressing RuBisCO and PRK [56, 57], expression levels of PRK in engineered *S. cerevisiae* strains needed to be ‘tuned’ to strike a balance between generating sufficient ribulose-1,5-bisphosphate for in vivo RuBisCO activity and avoiding negative effects of high-level PRK overexpression. Use of the medium-strength, anaerobically inducible *DAN1* promoter was shown to meet these requirements. An additional advantage of using an anaerobically inducible promoter for *prk* expression is that it minimizes any negative effects of PRK expression during the aerobic biomass propagation phase that precedes anaerobic industrial processes for bioethanol production [79].

In the original strain design, which carried a functional *GPD2* gene and in which the PPP enzymes were not overexpressed, the contribution of the engineered PRK/RuBisCO pathway to in vivo NADH oxidation was negatively correlated with specific growth rate. The effect of additional overexpression of the non-oxidative pentose-phosphate pathway genes *RPE1*, *TKL1*, *TAL1*, *NQM1*, *RKI1* and *TKL2* identified supply of ribulose-5-phosphate and/or other intermediates of the PPP as a key factor in the PRK/RuBisCO-mediated CO_2_ reduction in *S. cerevisiae*. Overexpression of non-oxidative PPP genes is a well-documented element in the construction of xylose- and arabinose-fermenting *S. cerevisiae* strains for fermentation of lignocellulosic hydrolysates [61, 80, 81], which should facilitate implementation of PRK/RuBisCO-enabled CO_2_ reduction in such strains. The positive effect of the deletion of *GPD2* on CO_2_ reduction is consistent with its reported beneficial effect on strains utilizing acetic acid as an external electron acceptor for redox cofactor balancing [22].

A mechanistic, quantitative understanding of the mechanisms by which glycerol formation and RuBisCO/PRK-mediated CO_2_ reduction interact in a growth-rate-dependent manner would require advanced analyses of intracellular metabolite concentrations in the yeast cytosol, which are beyond the scope of the present study. Clearly, the cytosolic NADH/NAD^+^ ratio, which affects regulation of *GPD2* [17] and is involved in the reductive reactions in both pathways, would be of special interest in such studies. In addition, the triose-phosphate node in glycolysis is of special interest, since Gpd1 and Gpd2 use dihydroxyacetone phosphate (DHAP) as a substrate, while the glycolytic intermediate glyceraldehyde-3-phosphate (GAP) is a substrate of transketolase, a key enzyme for provision of the RuBisCO substrate ribulose-5-phosphate via the non-oxidative PPP. Intracellular concentrations of DHAP in glucose-grown cultures of *S. cerevisiae* increase when the specific growth rate increases [82], presumably reflecting the higher glycolytic flux in fast-growing cultures. The equilibrium of the triose phosphate isomerase lies far towards DHAP [83], intracellular concentrations of which have been reported to be in the low mM range in *S. cerevisiae* [84]. Intracellular concentrations of GAP are therefore likely to be below the reported high *K*_*m*_ value of yeast transketolase for this substrate (ca. 5 mM, [85]), which may well contribute to the impact of overexpression of the PPP pathway on flux distribution at this branchpoint.

Whole genome duplications have been previously shown in *S. cerevisiae* strains obtained by evolutionary engineering [86, 87]. However, its occurrence in our initially constructed strains, which were only subjected to targeted, CRISPR-mediated genetic modification, was unexpected. Since 5-fluoro-orotic acid has been reported to affect chromosome segregation in yeasts [88], we cannot exclude the possibility that the genome duplication was related to counter-selection with 5-fluoro-orotic acid to recycle the gRNA-expressing plasmid during the construction of strain IMX765, the parental strain of IMX773 and IMX774. When using traditional genetic modification techniques, such as one-step gene replacement, aneuploidies or genome duplications are easily identified by simple diagnostic PCR experiments. However, the high efficiency of CRISPR/Cas9-based genome editing generally results in simultaneous modification of both copies of a target locus in diploid or aneuploid strains [89]. In this light, it is advisable to perform flow-cytometry-based ploidy analysis and whole genome sequencing to detect genome duplications and aneuploidy, respectively [65], when CRISPR technology is used for strain construction.

The characteristics of the engineered strains make this engineering strategy very interesting for further testing in industrial settings. In contrast to a previously published strategy for minimizing glycerol production by the reduction of acetic acid, by expression of a heterologous acetylating-acetaldehyde dehydrogenase in combination with the native alcohol dehydrogenases [20, 22], the current strategy does not require an organic electron acceptor. It is therefore compatible with fermentation of ‘first generation’ feedstocks that contain little or no acetic acid.

Research on production of alternative compatible solutes is ongoing, with trehalose production being a promising candidate, but so far glycerol remains the key metabolite involved in tolerance of sugar-grown *S. cerevisiae* cultures to osmotic stress [90–92]. The presence, in the CO_2_-reducing strains described in this study, of a functional *GPD1* gene was sufficient to maintain osmotolerance. At high osmolarity, upregulation of *GPD1* [16, 93] might reduce the stoichiometric benefits of CO_2_-fixation in RuBisCO/PRK-expressing yeast strains. It may be possible to prevent such an effect by promoter replacement of *GPD1* by lower-strength ones [59]. Alternatively, the entire *GPD1* gene may be replaced by a heterologous gene encoding an NADP^+^-linked glycerol-3-phosphate dehydrogenase, thereby uncoupling the roles of glycerol in redox homeostasis and osmotolerance [22].

## Conclusions

Overexpression of the Calvin-cycle enzymes, RuBisCO and PRK, in combination with deletion of *GPD2* and overexpression of the genes of the non-oxidative branch of the pentose-phosphate pathway, yielded *S. cerevisiae* strains that displayed a ca. 90% decrease in glycerol production and a 15% increase in ethanol yield on sugar, without affecting the maximum specific growth rate. Based on our experiments in synthetic media, the presented metabolic engineering strategy has the potential to enable significant improvements in the ethanol yields in industrial processes. The industrial application of this strategy should not require special process conditions or media compositions, and is ready for implementation in industrial strain backgrounds and subsequent evaluation in first- and second-generation industrial substrates.

## Additional files

**Additional file 1.** Primers used in this study.

**Additional file 2.** Organic acid production in anaerobic bioreactor batch and chemostat cultures of *S. cerevisiae* strains constructed in this study. Cultures were grown on synthetic medium containing 20 g L^−1^ glucose (pH 5). Values represent averages ± mean deviations of measurements taken at the end of the fermentations in the case of batch cultures, and during steady-state in chemostat cultures. Batch cultures of IME324 and IMX1443 were performed in triplicate. Batch cultures of IMX774 were performed in quadruplicate and cultures of all other strains were performed in duplicate.

**Additional file 3.** Osmotolerance assay of engineered strains. Cells were grown on synthetic medium (180 g L^−1^ (1M) glucose, initial pH 6) and incubated at 30 °C for 48h under anaerobic conditions (10% CO_2_). A: IME324 (*GPD1 GPD2*); B: IMX1443 (*GPD1 gpd2Δ* p*DAN1-prk cbbm* non-ox PPP↑, diploid); C: IMX1489 (*GPD1 gpd2Δ* p*DAN1-prk cbbm* non-ox PPP↑, haploid).

**Additional file 4.** Ploidy assessment of engineered RuBisCO/PRK-expressing strains. DNA content of each strain (blue) was measured by flow cytometric analysis and compared to the haploid strain CEN.PK113-5D (red; upper panel) and the diploid strain CEN.PK122 (red; bottom panel). A: IMX581 (*GPD1 GPD2*, parental of lineage); B: IMX765 (*GPD1 GPD2 cbbm*); C: IMX773 (*GPD1 GPD2* p*YEN1*-*prk cbbm*); D: IMX774 (*GPD1 GPD2* p*DAN1*-*prk cbbm*); E: IMX1489 (*GPD1 gpd2Δ* p*DAN1*-*prk cbbm* non-ox PPP↑).

**Additional file 5.** Sequence coverage plots comparing the genome of RuBisCO/PRK-expressing strain IMX774 to a published genome of CEN.PK113-7D [30], generated using BWA to map the sequence reads from IMX774 to the CEN.PK113-7D reference. Further processed by SAMtools to extract the per base sequence depth and an in-house script to calculate the average coverage for 500 bp non-overlapping windows. R script was used to plot the 500 bp windows (black dots) and median coverage (red line).

## Abbreviations

RuBisCO: ribulose-1,5-bisphosphate carboxylase
PRK: phosphoribulokinase
ORF: open reading frame
PPP: pentose-phosphate pathway
DHAP: dihydroxyacetone-phosphate
GAP: glyceraldehyde-3-phosphate

## References

[1] Hermann BG, Blok K, Patel MK (2007). Producing bio-based bulk chemicals using industrial biotechnology saves energy and combats climate change. Environ Sci Technol.

[2] Jansen MLA, Bracher JM, Papapetridis I, Verhoeven MD, de Bruijn H, de Waal PP, van Maris AJA, Klaassen P, Pronk JT (2017). *Saccharomyces cerevisiae* strains for second-generation ethanol production: from academic exploration to industrial implementation. FEMS Yeast Res.

[3] Della-Bianca BE, Basso TO, Stambuk BU, Basso LC, Gombert AK (2013). What do we know about the yeast strains from the Brazilian fuel ethanol industry?. Appl Microbiol Biotechnol.

[4] Nielsen J, Larsson C, van Maris AJA, Pronk JT (2013). Metabolic engineering of yeast for production of fuels and chemicals. Curr Opin Biotechnol.

[5] Gombert AK, van Maris AJA (2015). Improving conversion yield of fermentable sugars into fuel ethanol in 1st generation yeast-based production processes. Curr Opin Biotechnol.

[6] Lopes ML, de Lima Paulillo SC, Godoy A, Cherubin RA, Lorenzi MS, Giometti FHC, Bernardino CD, de Amorim Berbert, Neto H, Vianna de Amorim H (2016). Ethanol production in Brazil: a bridge between science and industry. Braz J Microbiol.

[7] Maiorella BL, Blanch HW, Wilke CR, Charles EWIB (2009). Economic evaluation of alternative ethanol fermentation processes. Biotechnol Bioeng.

[8] Nissen TL, Kielland-Brandt MC, Nielsen J, Villadsen J (2000). Optimization of ethanol production in *Saccharomyces cerevisiae* by metabolic engineering of the ammonium assimilation. Metab Eng.

[9] Van Dijken JP, Scheffers WA (1986). Redox balances in the metabolism of sugars by yeasts. FEMS Microbiol Lett.

[10] Verduyn C, Postma E, Scheffers WA, van Dijken JP (1990). Physiology of *Saccharomyces cerevisiae* in anaerobic glucose-limited chemostat cultures. J Gen Microbiol.

[11] Bishop WR, Bell RM (1988). Assembly of phospholipids into cellular membranes: biosynthesis, transmembrane movement and intracellular translocation. Ann Rev Cell Biol.

[12] Athenstaedt K, Daum G (2000). 1-Acyldihydroxyacetone-phosphate reductase (Ayr1p) of the yeast *Saccharomyces cerevisiae* encoded by the open reading frame *YIL124w* is a major component of lipid particles. J Biol Chem.

[13] Hohmann S (2002). Osmotic stress signaling and osmoadaptation in yeasts. Microbiol Mol Biol Rev.

[14] Nevoigt E, Stahl U (1997). Osmoregulation and glycerol metabolism in the yeast *Saccharomyces cerevisiae*. FEMS Microbiol Rev.

[15] Babazadeh R, Lahtvee P-J, Adiels CB, Goksör M, Nielsen JB, Hohmann S (2017). The yeast osmostress response is carbon source dependent. Sci Rep.

[16] Albertyn J, Hohmann S, Thevelein JM, Prior BA (1994). *GPD1*, which encodes glycerol-3-phosphate dehydrogenase, is essential for growth under osmotic stress in *Saccharomyces cerevisiae*, and its expression is regulated by the high-osmolarity glycerol response pathway. Mol Cell Biol.

[17] Ansell R, Granath K, Hohmann S, Thevelein JM, Adler L (1997). The two isoenzymes for yeast NAD^+^-dependent glycerol 3-phosphate dehydrogenase encoded by *GPD1* and *GPD2* have distinct roles in osmoadaptation and redox regulation. EMBO J.

[18] Larsson K, Ansell R, Eriksson P, Adler L (1993). A gene encoding sn-glycerol 3-phosphate dehydrogenase (NAD^+^) complements an osmosensitive mutant of *Saccharomyces cerevisiae*. Mol Microbiol.

[19] Björkqvist S, Ansell R, Adler L, Lidén G (1997). Physiological response to anaerobicity of glycerol-3-phosphate dehydrogenase mutants of *Saccharomyces cerevisiae*. Appl Environ Microbiol.

[20] Guadalupe-Medina V, Almering MJH, van Maris AJA, Pronk JT (2010). Elimination of glycerol production in anaerobic cultures of a *Saccharomyces cerevisiae* strain engineered to use acetic acid as an electron acceptor. Appl Environ Microbiol.

[21] Guadalupe-Medina V, Metz B, Oud B, van der Graaf CM, Mans R, Pronk JT, van Maris AJA (2014). Evolutionary engineering of a glycerol-3-phosphate dehydrogenase-negative, acetate-reducing *Saccharomyces cerevisiae* strain enables anaerobic growth at high glucose concentrations. Microb Biotechnol.

[22] Papapetridis I, van Dijk M, van Maris AJA, Pronk JT (2017). Metabolic engineering strategies for optimizing acetate reduction, ethanol yield and osmotolerance in *Saccharomyces cerevisiae*. Biotechnol Biofuels.

[23] Klinke HB, Thomsen AB, Ahring BK (2004). Inhibition of ethanol-producing yeast and bacteria by degradation products produced during pre-treatment of biomass. Appl Microbiol Biotechnol.

[24] Palmqvist E, Hahn-Hägerdal B (2000). Fermentation of lignocellulosic hydrolysates. II: inhibitors and mechanisms of inhibition. Bioresour Technol.

[25] Bro C, Regenberg B, Förster J, Nielsen J (2006). In silico aided metabolic engineering of *Saccharomyces cerevisiae* for improved bioethanol production. Metab Eng.

[26] Palmqvist E, Grage H, Meinander NQ, Hahn-Hägerdal B (1999). Main and interaction effects of acetic acid, furfural, and p-hydroxybenzoic acid on growth and ethanol productivity of yeasts. Biotechnol Bioeng.

[27] Taherzadeh MJ, Niklasson C, Lidén G (1997). Acetic acid—friend or foe in anaerobic batch conversion of glucose to ethanol by *Saccharomyces cerevisiae*?. Chem Eng Sci.

[28] Guadalupe-Medina V, Wisselink HW, Luttik MA, de Hulster E, Daran J-MG, Pronk JT, van Maris AJA (2013). Carbon dioxide fixation by Calvin-cycle enzymes improves ethanol yield in yeast. Biotechnol Biofuels.

[29] Entian K-D, Kötter P (2007). 25 yeast genetic strain and plasmid collections. Methods Microbiol..

[30] Nijkamp JF, van den Broek M, Datema E, de Kok S, Bosman L, Luttik MA, Daran-Lapujade P, Vongsangnak W, Nielsen J, Heijne WH (2012). De novo sequencing, assembly and analysis of the genome of the laboratory strain *Saccharomyces cerevisiae* CEN.PK113-7D, a model for modern industrial biotechnology. Microb Cell Fact.

[31] Verduyn C, Postma E, Scheffers WA, van Dijken JP (1992). Effect of benzoic acid on metabolic fluxes in yeasts: a continuous-culture study on the regulation of respiration and alcoholic fermentation. Yeast.

[32] DiCarlo JE, Norville JE, Mali P, Rios X, Aach J, Church GM (2013). Genome engineering in *Saccharomyces cerevisiae* using CRISPR-Cas systems. Nucleic Acids Res.

[33] Mikkelsen MD, Buron LD, Salomonsen B, Olsen CE, Hansen BG, Mortensen UH, Halkier BA (2012). Microbial production of indolylglucosinolate through engineering of a multi-gene pathway in a versatile yeast expression platform. Metab Eng.

[34] Mans R, van Rossum HM, Wijsman M, Backx A, Kuijpers NGA, van den Broek M, Daran-Lapujade P, Pronk JT, van Maris AJA, Daran J-MG (2015). CRISPR/Cas9: a molecular Swiss army knife for simultaneous introduction of multiple genetic modifications in *Saccharomyces cerevisiae*. FEMS Yeast Res.

[35] Knijnenburg TA, Daran J-MG, van den Broek MA, Daran-Lapujade P, de Winde JH, Pronk JT, Reinders MJT, Wessels LFA (2009). Combinatorial effects of environmental parameters on transcriptional regulation in *Saccharomyces cerevisiae*: a quantitative analysis of a compendium of chemostat-based transcriptome data. BMC Genom.

[36] Daniel Gietz R, Woods RA (2002). Transformation of yeast by lithium acetate/single-stranded carrier DNA/polyethylene glycol method. Methods Enzymol.

[37] Solis-Escalante D, Kuijpers NGA, Bongaerts N, Bolat I, Bosman L, Pronk JT, Daran J-MG, Daran-Lapujade P (2013). *amdSYM*, a new dominant recyclable marker cassette for *Saccharomyces cerevisiae*. FEMS Yeast Res.

[38] Mumberg D, Müller R, Funk M (1995). Yeast vectors for the controlled expression of heterologous proteins in different genetic backgrounds. Gene.

[39] Papapetridis I, van Dijk M, Dobbe AP, Metz B, Pronk JT, van Maris AJA (2016). Improving ethanol yield in acetate-reducing *Saccharomyces cerevisiae* by cofactor engineering of 6-phosphogluconate dehydrogenase and deletion of *ALD6*. Microb Cell Fact.

[40] Mashego MR, van Gulik WM, Vinke JL, Heijnen JJ (2003). Critical evaluation of sampling techniques for residual glucose determination in carbon-limited chemostat culture of *Saccharomyces cerevisiae*. Biotechnol Bioeng.

[41] Heijnen JJ, van Dijken JP (1992). In search of a thermodynamic description of biomass yields for the chemotrophic growth of microorganisms. Biotechnol Bioeng.

[42] Postma E, Verduyn C, Scheffers WA, van Dijken JP (1989). Enzymic analysis of the crabtree effect in glucose-limited chemostat cultures of *Saccharomyces cerevisiae*. Appl Environ Microbiol.

[43] MacElroy RD, Mack HM, Johnson EJ (1972). Properties of phosphoribulokinase from *Thiobacillus neapolitanus*. J Bacteriol.

[44] Lowry OH, Rosebrough NJ, Farr AL, Randall RJ (1951). Protein measurement with the folin phenol reagent. J Biol Chem.

[45] Van Rossum HM, Kozak BU, Niemeijer MS, Dykstra JC, Luttik MAH, Daran J-MG, van Maris AJA, Pronk JT (2016). Requirements for carnitine shuttle-mediated translocation of mitochondrial acetyl moieties to the yeast cytosol. mBio.

[46] Haase S, Reed S (2002). Improved flow cytometric analysis of the budding yeast cell cycle. Cell Cycle.

[47] Bracher JM, de Hulster E, Koster CC, van den Broek M, Daran J-MG, van Maris AJA, Pronk JT (2017). Laboratory evolution of a biotin-requiring *Saccharomyces cerevisiae* strain for full biotin prototrophy and identification of causal mutations. Appl Environ Microbiol.

[48] Verhoeven MD, Lee M, Kamoen L, van den Broek M, Janssen DB, Daran J-MG, van Maris AJA, Pronk JT (2017). Mutations in *PMR1* stimulate xylose isomerase activity and anaerobic growth on xylose of engineered *Saccharomyces cerevisiae* by influencing manganese homeostasis. Sci Rep.

[49] Walker BJ, Abeel T, Shea T, Priest M, Abouelliel A, Sakthikumar S, Cuomo CA, Zeng Q, Wortman J, Young SK, Earl AM (2014). Pilon: an integrated tool for comprehensive microbial variant detection and genome assembly improvement. PLoS ONE.

[50] Li H, Durbin R (2010). Fast and accurate long-read alignment with Burrows-Wheeler transform. Bioinformatics.

[51] Li H, Handsaker B, Wysoker A, Fennell T, Ruan J, Homer N, Marth G, Abecasis G, Durbin R, Genome Project Data Processing Subgroup (2009). The sequence alignment/map format and SAMtools. Bioinformatics.

[52] Van den Brink J, Akeroyd M, van der Hoeven R, Pronk JT, de Winde JH, Daran-Lapujade P (2009). Energetic limits to metabolic flexibility: responses of *Saccharomyces cerevisiae* to glucose–galactose transitions. Microbiology.

[53] Pronk JT (2002). Auxotrophic yeast strains in fundamental and applied research. App Environ Microbiol.

[54] Da Silva NA, Srikrishnan S (2012). Introduction and expression of genes for metabolic engineering applications in *Saccharomyces cerevisiae*. FEMS Yeast Res.

[55] Shi S, Liang Y, Zhang MM, Ang EL, Zhao H (2016). A highly efficient single-step, markerless strategy for multi-copy chromosomal integration of large biochemical pathways in *Saccharomyces cerevisiae*. Metab Eng.

[56] Parikh MR, Greene DN, Woods KK, Matsumura I (2006). Directed evolution of RuBisCO hypermorphs through genetic selection in engineered *E.coli*. Protein Eng Des Sel.

[57] Hudson G, Morell M, Arvidsson Y, Andrews T (1992). Synthesis of spinach phosphoribulokinase and ribulose 1,5-bisphosphate in *Escherichia coli*. Funct Plant Biol.

[58] Snoep JL, Yomano LP, Westerhoff HV, Ingram LO (1995). Protein burden in *Zymomonas mobilis*: negative flux and growth control due to overproduction of glycolytic enzymes. Microbiology.

[59] Hubmann G, Guillouet S, Nevoigt E (2011). Gpd1 and Gpd2 fine-tuning for sustainable reduction of glycerol formation in *Saccharomyces cerevisiae*. Appl Environ Microbiol.

[60] Nissen TL, Hamann CW, Kielland-Brandt MC, Nielsen J, Villadsen J (2000). Anaerobic and aerobic batch cultivations of *Saccharomyces cerevisiae* mutants impaired in glycerol synthesis. Yeast.

[61] Kuyper M, Hartog MMP, Toirkens MJ, Almering MJH, Winkler AA, van Dijken JP, Pronk JT (2005). Metabolic engineering of a xylose-isomerase-expressing *Saccharomyces cerevisiae* strain for rapid anaerobic xylose fermentation. FEMS Yeast Res.

[62] Wisselink HW, Cipollina C, Oud B, Crimi B, Heijnen JJ, Pronk JT, van Maris AJA (2010). Metabolome, transcriptome and metabolic flux analysis of arabinose fermentation by engineered *Saccharomyces cerevisiae*. Metab Eng.

[63] Walfridsson M, Hallborn J, Penttilä M, Keränen S, Hahn-Hägerdal B (1995). Xylose-metabolizing *Saccharomyces cerevisiae* strains overexpressing the *TKL1* and *TAL1* genes encoding the pentose phosphate pathway enzymes transketolase and transaldolase. Appl Environ Microbiol.

[64] Karhumaa K, Hahn-Hägerdal B, Gorwa-Grauslund M-F (2005). Investigation of limiting metabolic steps in the utilization of xylose by recombinant *Saccharomyces cerevisiae* using metabolic engineering. Yeast.

[65] de Vries AR, Pronk JT, Daran J-MG (2017). Industrial relevance of chromosomal copy number variation in *Saccharomyces* yeasts. Appl Environ Microbiol.

[66] Calvin M, Benson AA (1948). The path of carbon in photosynthesis. Science.

[67] Ducat DC, Silver PA (2012). Improving carbon fixation pathways. Curr Opin Chem Biol.

[68] Lin MT, Occhialini A, Andralojc PJ, Parry MAJ, Hanson MR (2014). A faster RubisCO with potential to increase photosynthesis in crops. Nature.

[69] McKinlay JB, Harwood CS (2010). Carbon dioxide fixation as a central redox cofactor recycling mechanism in bacteria. PNAS.

[70] Gong F, Liu G, Zhai X, Zhou J, Cai Z, Li Y (2015). Quantitative analysis of an engineered CO_2_-fixing *Escherichia coli* reveals great potential of heterotrophic CO_2_ fixation. Biotechnol Biofuels.

[71] Bischoff KM, Liu S, Leathers TD, Worthington RE, Rich JO (2009). Modeling bacterial contamination of fuel ethanol fermentation. Biotechnol Bioeng.

[72] Jain VK, Divol B, Prior BA, Bauer FF (2011). Elimination of glycerol and replacement with alternative products in ethanol fermentation by *Saccharomyces cerevisiae*. J Ind Microbiol Biotechnol.

[73] Wu G, Yan Q, Jones JA, Tang YJ, Fong SS, Koffas MAG (2016). Metabolic burden: cornerstones in synthetic biology and metabolic engineering applications. Trends Biotechnol.

[74] Aigner H, Wilson RH, Bracher A, Calisse L, Bhat JY, Hartl FU, Hayer-Hartl M (2017). Plant RuBisCo assembly in *E. coli* with five chloroplast chaperones including BSD2. Science.

[75] Temer B, dos Santos LV, Negri VA, Galhardo JP, Magalhães PHM, José J, Marschalk C, Corrêa TLR, Carazzolle MF, Pereira GAG (2017). Conversion of an inactive xylose isomerase into a functional enzyme by co-expression of GroEL-GroES chaperonins in *Saccharomyces cerevisiae*. BMC Biotechnol.

[76] Jariyachawalid K, Laowanapiban P, Meevootisom V, Wiyakrutta S (2012). Effective enhancement of *Pseudomonas stutzeri*d-phenylglycine aminotransferase functional expression in *Pichia pastoris* by co-expressing *Escherichia coli* GroEL-GroES. Microb Cell Fact.

[77] Xia PF, Turner TL, Jayakody LN (2016). The role of GroE chaperonins in developing biocatalysts for biofuel and chemical production. Enz Eng.

[78] Li Y-J, Wang M-M, Chen Y-W, Wang M, Fan L-H, Tan T-W (2017). Engineered yeast with a CO_2_-fixation pathway to improve the bio-ethanol production from xylose-mixed sugars. Sci Rep.

[79] Bellissimi E, Ingledew WM (2005). Metabolic acclimatization: preparing active dry yeast for fuel ethanol production. Process Biochem.

[80] Wisselink HW, Toirkens MJ, del Rosario Franco Berriel M, Winkler AA, Dijken JP, Pronk JT, van Maris AJA (2007). Engineering of *Saccharomyces cerevisiae* for efficient anaerobic alcoholic fermentation of l-arabinose. App. Environ Microbiol.

[81] Becker J, Boles E (2003). A modified *Saccharomyces cerevisiae* strain that consumes l-arabinose and produces ethanol. App Environ Microbiol.

[82] Boer VM, Crutchfield CA, Bradley PH, Botstein D, Rabinowitz JD (2010). Growth-limiting intracellular metabolites in yeast growing under diverse nutrient limitations. Mol Biol Cell.

[83] Nickbarg EB, Knowles JR (1988). Triosephosphate isomerase: energetics of the reaction catalyzed by the yeast enzyme expressed in *Escherichia coli*. Biochemistry.

[84] Christen S, Sauer U (2011). Intracellular characterization of aerobic glucose metabolism in seven yeast species by ^13^C flux analysis and metabolomics. FEMS Yeast Res.

[85] Schenk G, Duggleby RG, Nixon PF (1998). Properties and functions of the thiamin diphosphate dependent enzyme transketolase. Int J Biochem Cell Biol.

[86] Oud B, Guadalupe-Medina V, Nijkamp JF, De Ridder D, Pronk JT, van Maris AJA, Daran J-MG (2013). Genome duplication and mutations in *ACE2* cause multicellular, fast-sedimenting phenotypes in evolved *Saccharomyces cerevisiae*. PNAS.

[87] Voordeckers K, Kominek J, Das A, Espinosa-Cantú A, De Maeyer D, Arslan A, Van Pee M, van der Zande E, Meert W, Yang Y (2015). Adaptation to high ethanol reveals complex evolutionary pathways. PLoS Genet.

[88] Wellington M, Rustchenko E (2005). 5-fluoro-orotic acid induces chromosome alterations in *Candida albicans*. Yeast.

[89] Stovicek V, Borodina I, Forster J (2015). CRISPR—Cas system enables fast and simple genome editing of industrial *Saccharomyces cerevisiae* strains. Metab Eng Commun.

[90] Shen B, Hohmann S, Jensen RG, Bohnert HJ (1999). Roles of sugar alcohols in osmotic stress adaptation. Replacement of glycerol by mannitol and sorbitol in yeast. Plant Physiol.

[91] Hounsa C-G, Brandt EV, Thevelein J, Hohmann S, Prior BA (1998). Role of trehalose in survival of *Saccharomyces cerevisiae* under osmotic stress. Microbiology.

[92] Guo Z-P, Zhang L, Ding Z-Y, Shi G-Y (2011). Minimization of glycerol synthesis in industrial ethanol yeast without influencing its fermentation performance. Metab Eng.

[93] Hirayama T, Maeda T, Saito H, Shinozaki K (1995). Cloning and characterization of seven cDNAs for hyperosmolarity-responsive (HOR) genes of *Saccharomyces cerevisiae*. Mol Gen Genet.

