# Supplementary material for: Optimizing anaerobic growth rate and fermentation kinetics in Saccharomyces cerevisiae strains expressing Calvin-cycle enzymes for improved ethanol yield
Source: Biotechnol Biofuels. 2018 Jan 25;11:17. doi: 10.1186/s13068-017-1001-z (PMC5784725; doi:10.1186/s13068-017-1001-z)
Supplement: Supplementary file 2 — Additional file 2. Organic acid production in anaerobic bioreactor batch and chemostat cultures of S. cerevisiae strains constructed in this study. Cultures were grown on synthetic medium containing 20 g L−1 glucose (pH 5). Values represent averages ± mean deviations of measurements taken at the end of the fermentations in the case of batch cultures, and during steady-state in chemostat cultures. Batch cultures of IME324 and IMX1443 were performed in triplicate. Batch cultures of IMX774 were performed in quadruplicate and cultures of all other strains were performed in duplicate. [file 13068_2017_1001_MOESM2_ESM.docx]

Additional File 2.

| Culture mode | Strain name | Acetate (mM) | Pyruvate (mM) | Succinate (mM) | Lactate (mM) |
| --- | --- | --- | --- | --- | --- |
| Batch | IME324 | 2.79 ± 0.11 | 0.71 ± 0.02 | 0.80 ± 0.05 | 2.01 ± 0.07 |
|  | IME369 | 2.71 ± 0.58 | 0.61 ± 0.06 | 0.59 ± 0.01 | 1.71 ± 0.18 |
|  | IMX773 | 2.63 ± 0.14 | 0.57 ± 0.01 | 1.12 ± 0.03 | 1.79 ± 0.02 |
|  | IMX774 | 4.17 ± 0.48 | 0.86 ± 0.10 | 0.76 ± 0.07 | 1.65 ± 0.14 |
|  | IMX949 | 1.99 ± 0.06 | 0.91 ± 0.01 | 0.65 ± 0.01 | 1.97 ± 0.01 |
|  | IMX1443 | 4.13 ± 0.18 | 0.81 ± 0.02 | 0.88 ± 0.06 | 1.99 ± 0.13 |
|  | IMX1489 | 4.60 ± 0.30 | 0.90 ± 0.00 | 0.83 ± 0.06 | 1.98 ± 0.06 |
| Chemostat D = 0.05 h^-1^ | IME324 | 0.55 ± 0.11 | 0.12 ± 0.15 | 0.92 ± 0.03 | 1.06 ± 0.25 |
| Chemostat D = 0.05 h^-1^ | IMX774 | 2.46 ± 0.31 | 0.27 ± 0.08 | 0.82 ± 0.00 | 1.28 ± 0.01 |
| Chemostat D = 0.15 h^-1^ | IME324 | 0.71 ± 0.18 | 0.40 ± 0.07 | 0.79 ± 0.01 | 1.80 ± 0.08 |
| Chemostat D = 0.15 h^-1^ | IMX774 | 1.75 ± 0.16 | 0.60 ± 0.04 | 1.07 ± 0.04 | 1.76 ± 0.02 |
