# Supplementary figures and images for: Optimizing anaerobic growth rate and fermentation kinetics in Saccharomyces cerevisiae strains expressing Calvin-cycle enzymes for improved ethanol yield
Source: Biotechnol Biofuels. 2018 Jan 25;11:17. doi: 10.1186/s13068-017-1001-z (PMC5784725; doi:10.1186/s13068-017-1001-z)

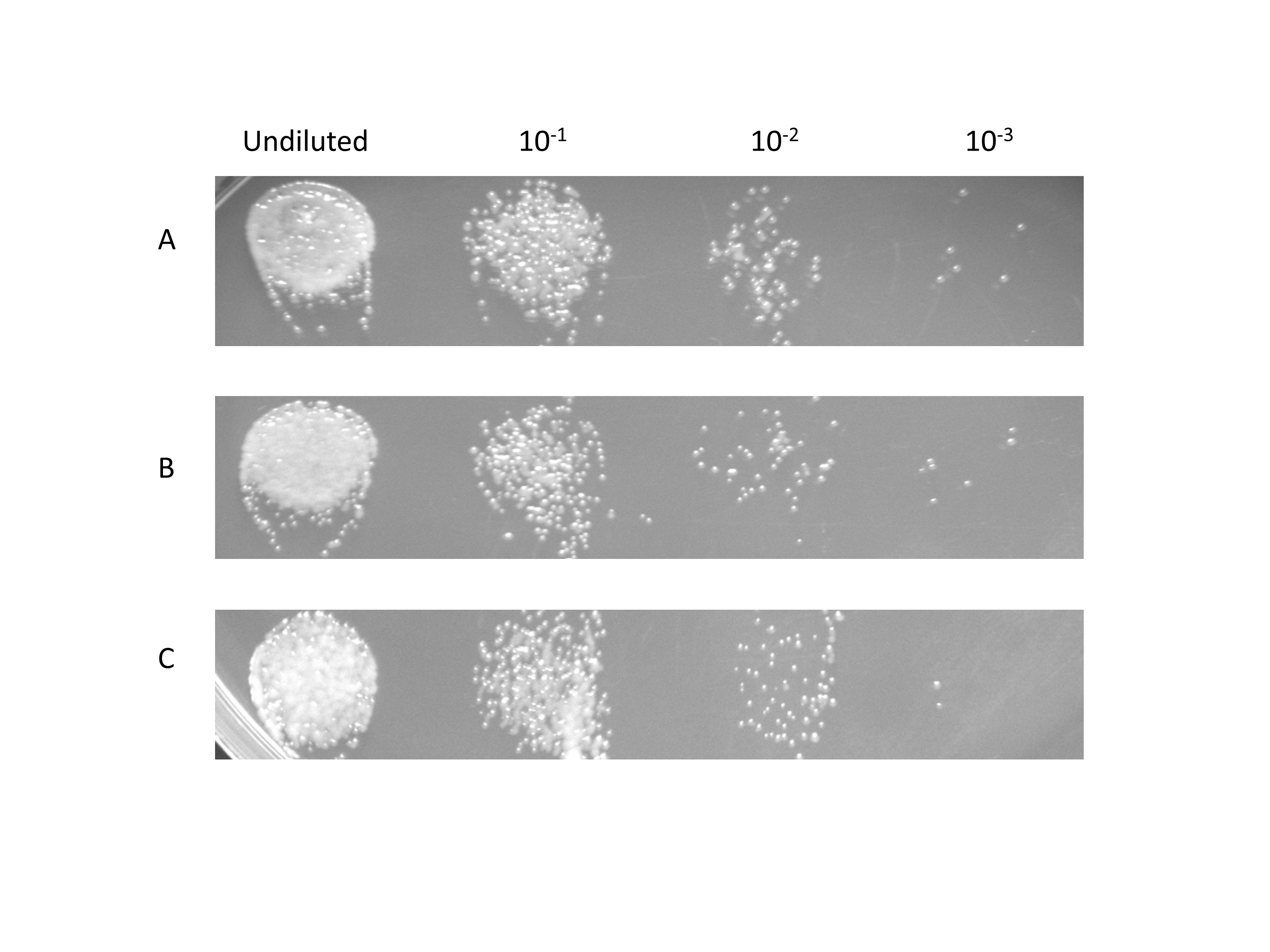

Supplement: Supplementary file 3 — Additional file 3. Osmotolerance assay of engineered strains. Cells were grown on synthetic medium (180 g L−1 (1M) glucose, initial pH 6) and incubated at 30 °C for 48h under anaerobic conditions (10% CO2). A: IME324 (GPD1 GPD2); B: IMX1443 (GPD1 gpd2Δ pDAN1-prk cbbm non-ox PPP↑, diploid); C: IMX1489 (GPD1 gpd2Δ pDAN1-prk cbbm non-ox PPP↑, haploid). [file 13068_2017_1001_MOESM3_ESM.tif]

Additional File 5.


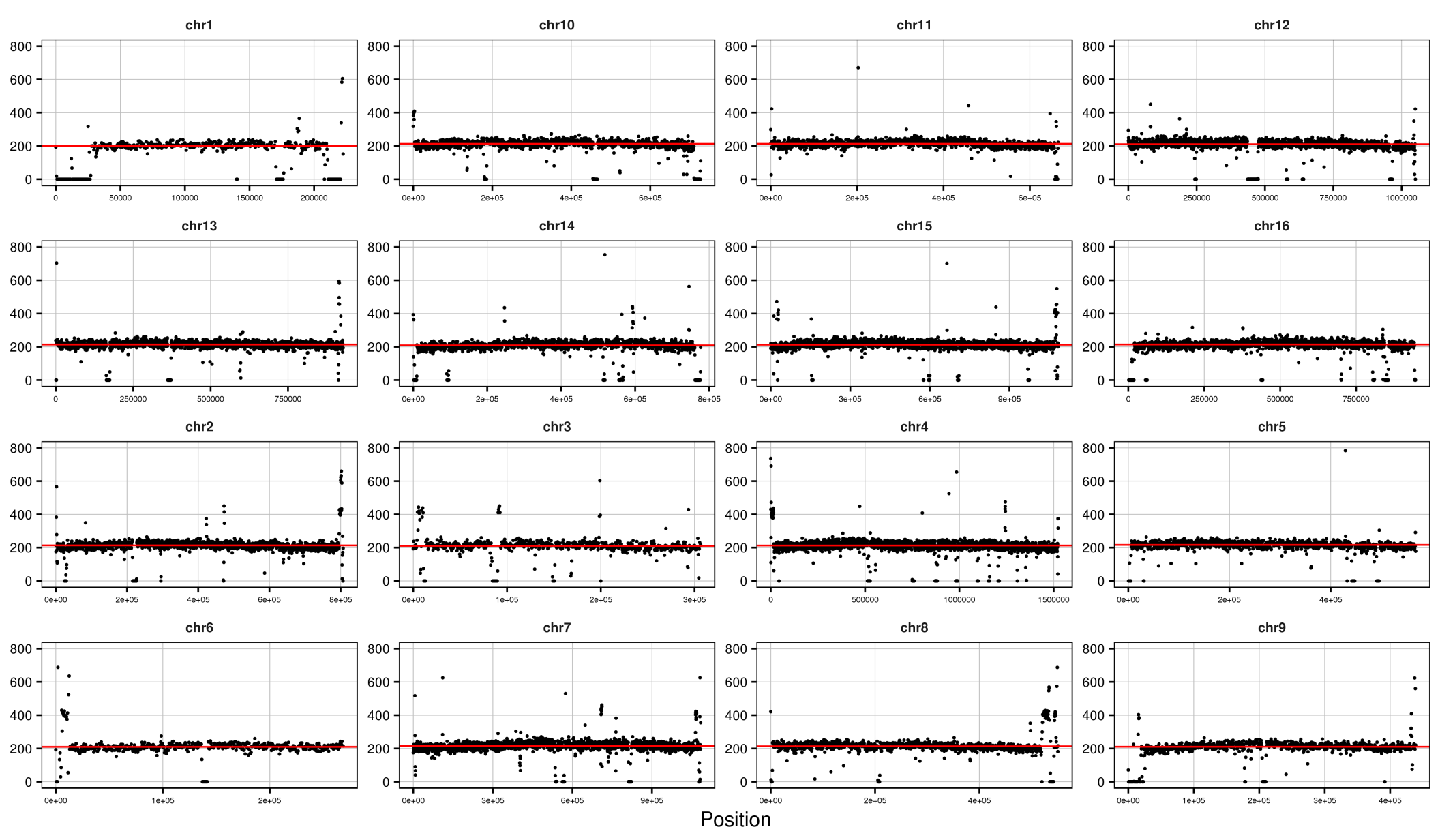

Supplement: Supplementary file 5 — Additional file 5. Sequence coverage plots comparing the genome of RuBisCO/PRK-expressing strain IMX774 to a published genome of CEN.PK113-7D [30], generated using BWA to map the sequence reads from IMX774 to the CEN.PK113-7D reference. Further processed by SAMtools to extract the per base sequence depth and an in-house script to calculate the average coverage for 500 bp non-overlapping windows. R script was used to plot the 500 bp windows (black dots) and median coverage (red line). [file 13068_2017_1001_MOESM5_ESM.docx]
