# Supplementary material for: Optimizing anaerobic growth rate and fermentation kinetics in Saccharomyces cerevisiae strains expressing Calvin-cycle enzymes for improved ethanol yield
Source: Biotechnol Biofuels. 2018 Jan 25;11:17. doi: 10.1186/s13068-017-1001-z (PMC5784725; doi:10.1186/s13068-017-1001-z)
Supplement: Supplementary file 4 — Additional file 4. Ploidy assessment of engineered RuBisCO/PRK-expressing strains. DNA content of each strain (blue) was measured by flow cytometric analysis and compared to the haploid strain CEN.PK113-5D (red; upper panel) and the diploid strain CEN.PK122 (red; bottom panel). A: IMX581 (GPD1 GPD2, parental of lineage); B: IMX765 (GPD1 GPD2 cbbm); C: IMX773 (GPD1 GPD2 pYEN1-prk cbbm); D: IMX774 (GPD1 GPD2 pDAN1-prk cbbm); E: IMX1489 (GPD1 gpd2Δ pDAN1-prk cbbm non-ox PPP↑). [file 13068_2017_1001_MOESM4_ESM.pdf]

A

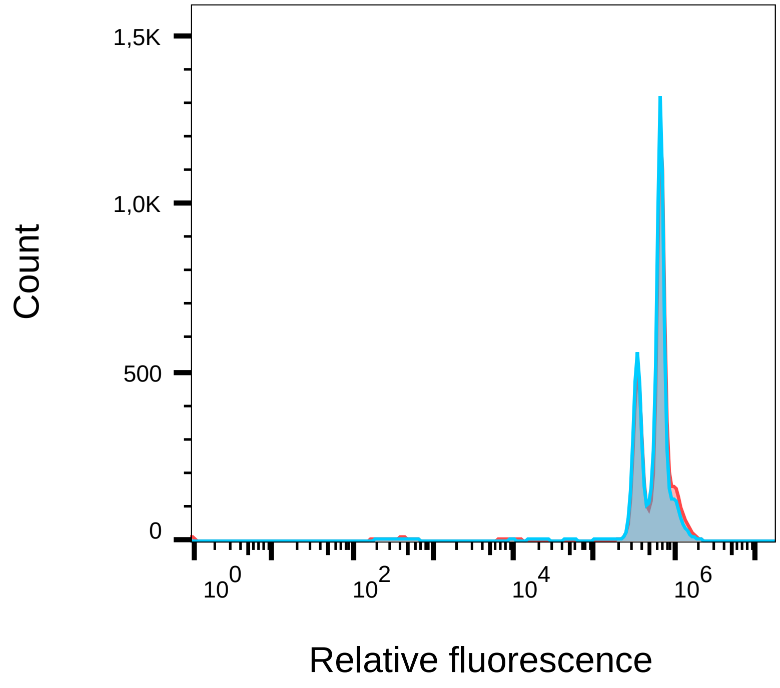

	Sample Name	Subset Name	Count
(cyan)	A03 581.fcs	Ungated	10000
(red)	A01 5D.fcs	Ungated	10000

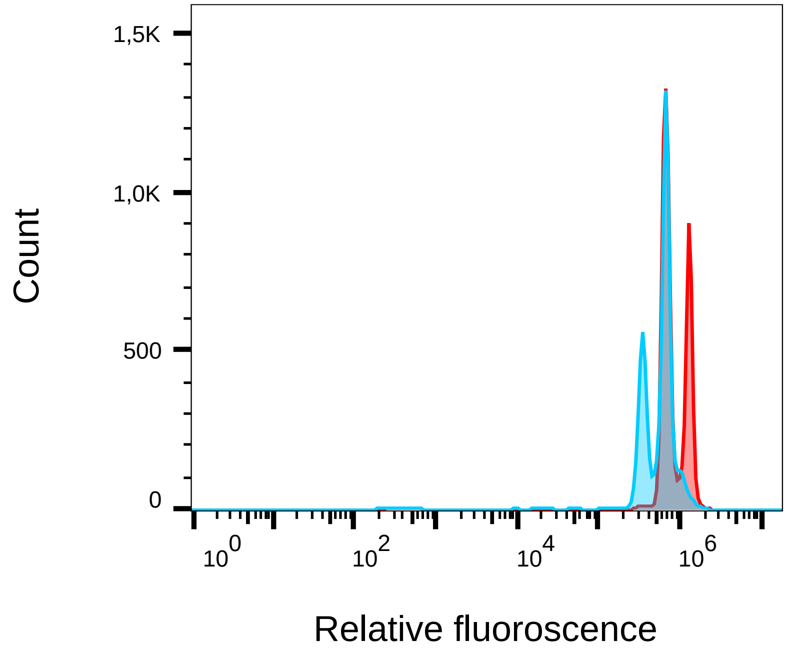

	Sample Name	Subset Name	Count
(cyan)	A03 581.fcs	Ungated	10000
(red)	A02 122.fcs	Ungated	10000

B

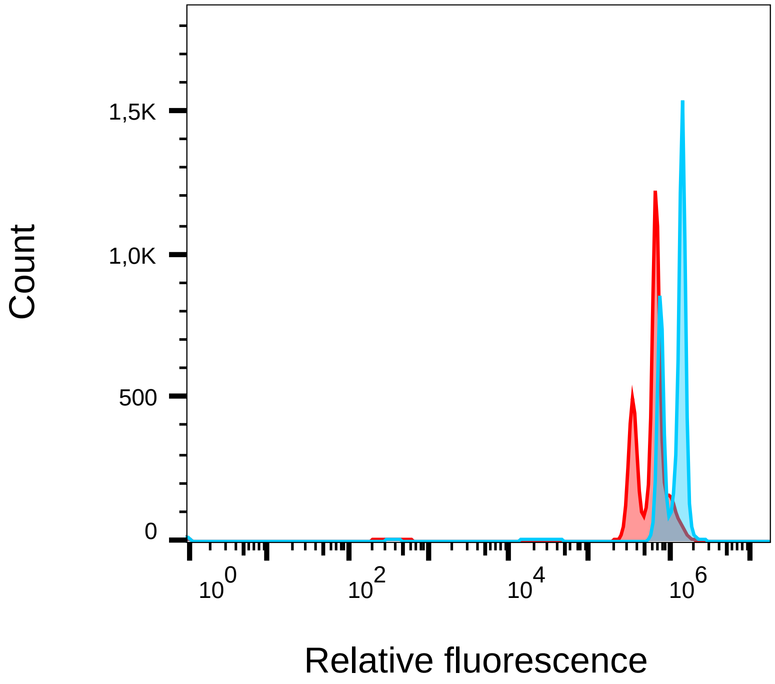

	Sample Name	Subset Name	Count
	A04 IMX765.fcs	Ungated	10000
	A01 5D.fcs	Ungated	10000

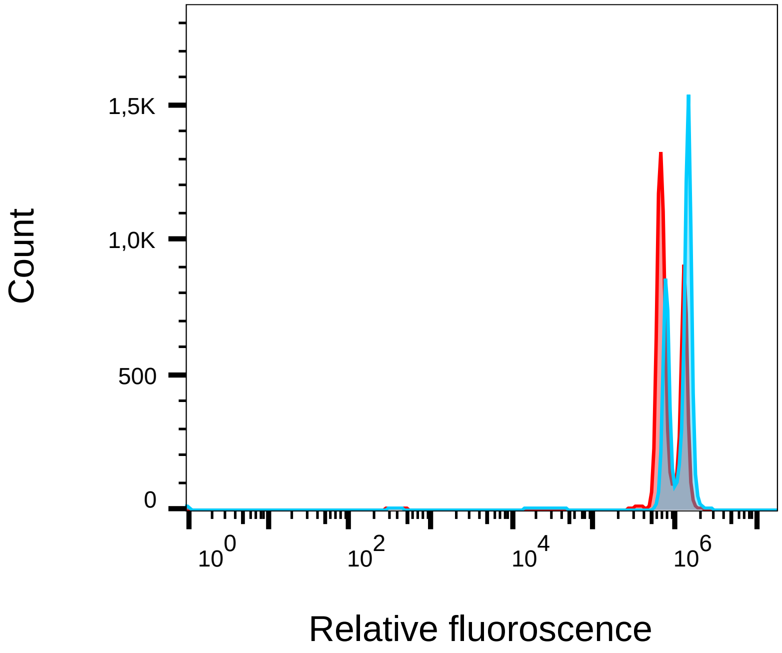

	Sample Name	Subset Name	Count
	A04 IMX765.fcs	Ungated	10000
	A02 122.fcs	Ungated	10000

C

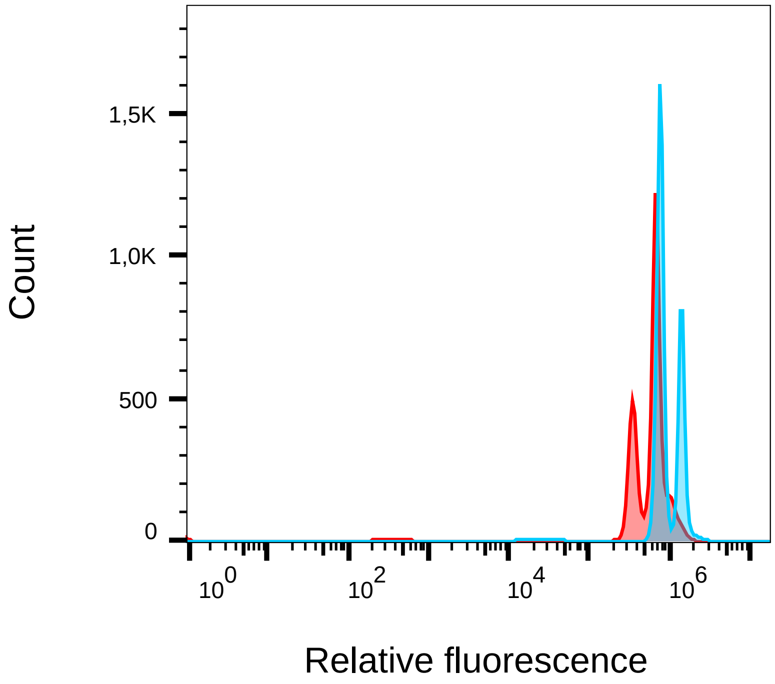

	Sample Name	Subset Name	Count
(cyan)	A05 IMX773.fcs	Ungated	10000
(red)	A01 5D.fcs	Ungated	10000

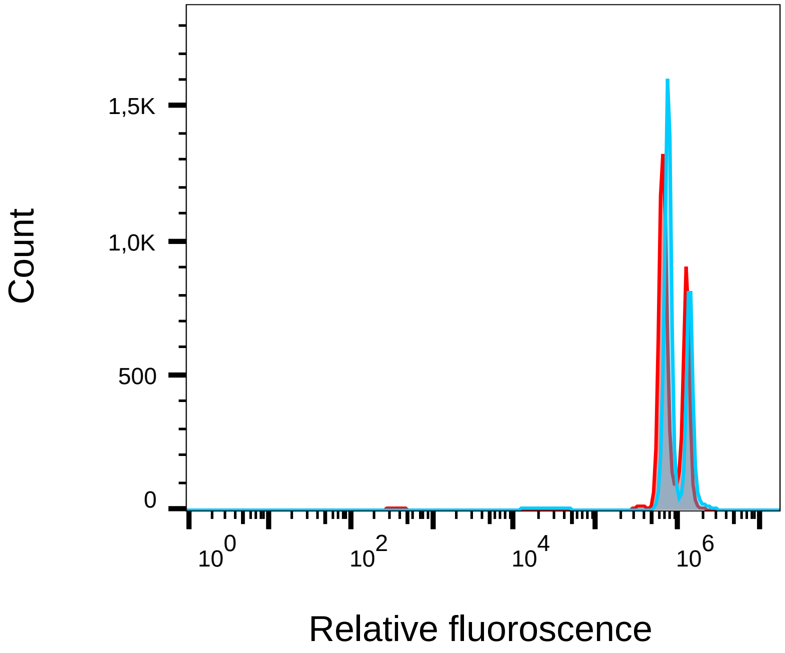

	Sample Name	Subset Name	Count
(cyan)	A05 IMX773.fcs	Ungated	10000
(red)	A02 122.fcs	Ungated	10000

D

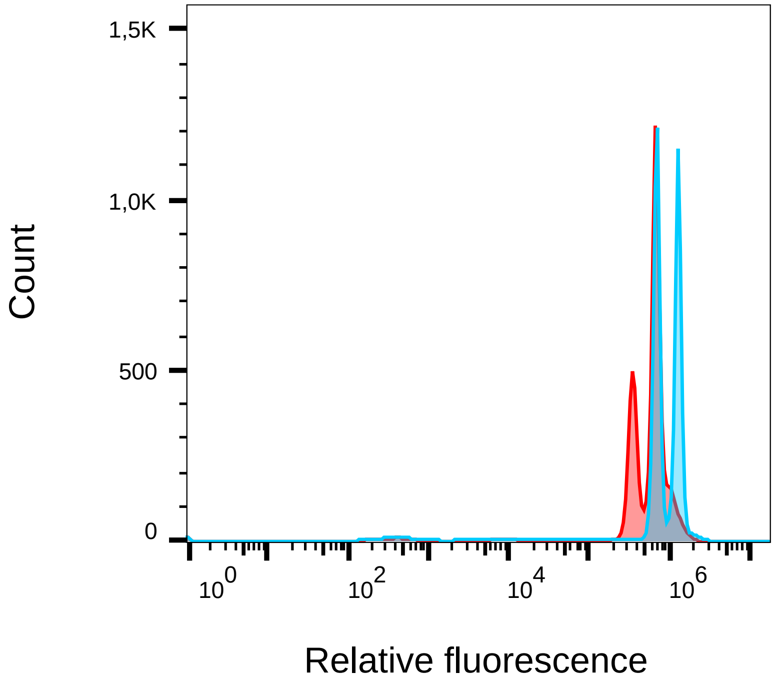

	Sample Name	Subset Name	Count
■ (cyan)	A04 774.fcs	Ungated	10000
■ (red)	A01 5D.fcs	Ungated	10000

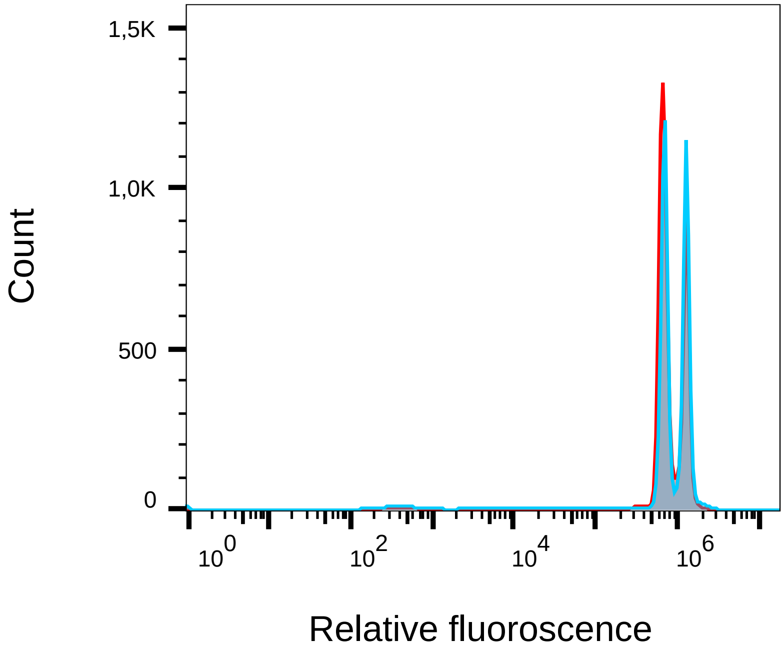

	Sample Name	Subset Name	Count
■ (cyan)	A04 774.fcs	Ungated	10000
■ (red)	A02 122.fcs	Ungated	10000

E

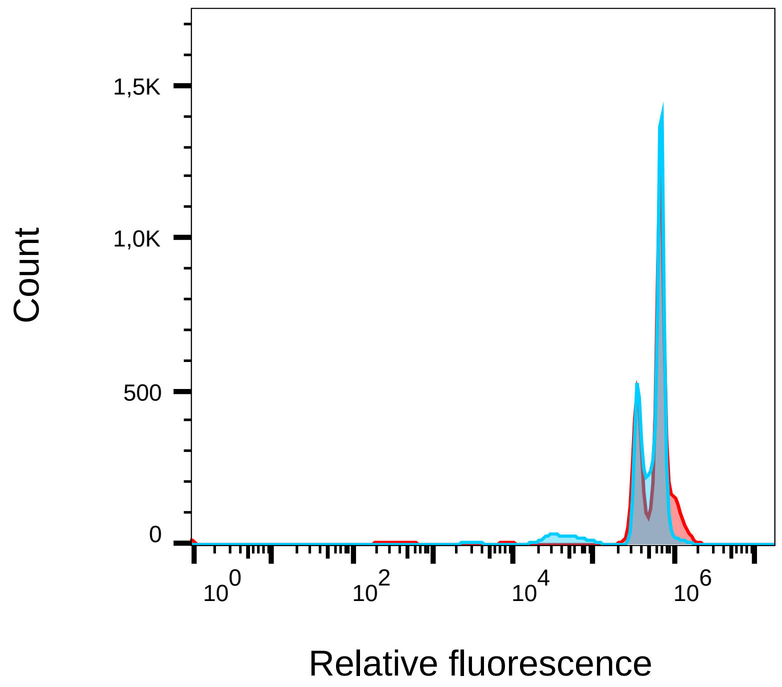

	Sample Name	Subset Name	Count
■ (blue)	A03 IMX1489.fcs	Ungated	10000
■ (red)	A01 5D.fcs	Ungated	10000

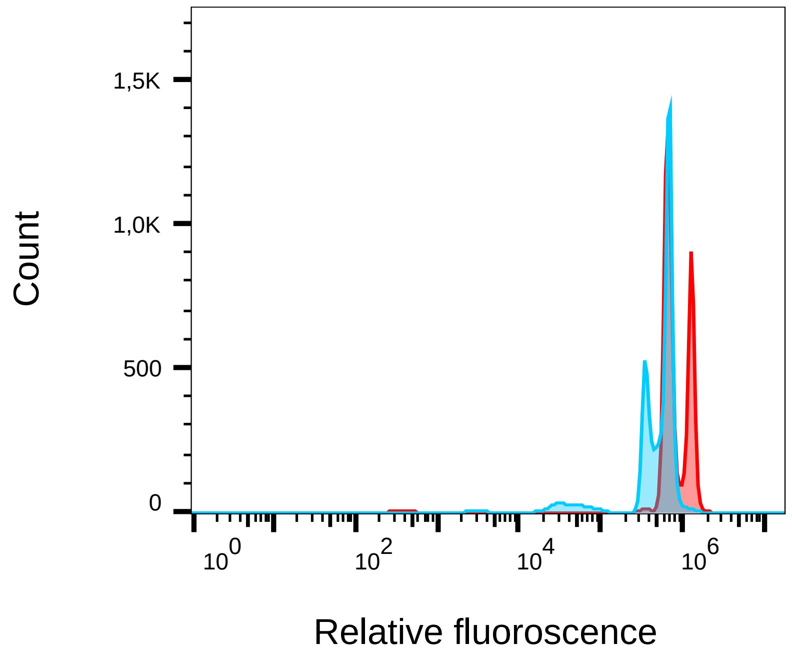

	Sample Name	Subset Name	Count
■ (blue)	A03 IMX1489.fcs	Ungated	10000
■ (red)	A02 122.fcs	Ungated	10000
